# TRIM26 inhibited osteosarcoma progression through destabilizing RACK1 and thus inactivation of MEK/ERK signaling

**DOI:** 10.1038/s41419-023-06048-9

**Published:** 2023-08-17

**Authors:** Kezhou Xia, Di Zheng, Zhun Wei, Wenda Liu, Weichun Guo

**Affiliations:** Department of Orthopedics, Renmin Hospital of Wuhan University, Wuhan, 430060 China

**Keywords:** Bone cancer, Tumour-suppressor proteins

## Abstract

Osteosarcoma is a highly aggressive malignant tumor that is common in the pediatric population and has a high rate of disability and mortality. Recent studies have suggested that the tripartite motif-containing family genes (TRIMs) play critical roles in oncogenesis in several cancers. TRIM26, one of the TRIMs family genes, was more frequently reported to exert a tumor-suppressive role, while its detailed functional roles in the osteosarcoma progression were still unknown and require further investigation. Herein, we found that TRIM26 was markedly downregulated in osteosarcoma tissues and cells. Survival analysis revealed that higher expression of TRIM26 was associated with better prognosis and its expression was an independent protective factor in osteosarcoma. Functional analysis demonstrated that overexpression of TRIM26 inhibited osteosarcoma cell proliferation and invasion via inhibiting the EMT process and MEK/ERK signaling. In contrast, the silence of TRIM26 caused the opposite effect. RACK1, a member of the Trp-Asp repeat protein family, was identified as a novel target of TRIM26. TRIM26 could interact with RACK1 and accelerate the degradation of RACK1, thus inactivation of MEK/ERK signaling. Overexpression of RACK1 could attenuate the inhibitory effect of TRIM26 overexpression on p-MEK1/2 and p-ERK1/2, and silence of RACK1 could partly impair the effect of TRIM26 knockdown-induced upregulation of p-MEK1/2 and p-ERK1/2. Further, a series of gain- and loss-of-function experiments showed that decreased malignant behaviors including cell proliferation and invasion in TRIM26-upregulated cells were reversed when RACK1 was overexpressed, whereas RACK1 knockdown diminished the increased malignant phenotypes in TRIM26-silenced osteosarcoma cells. In conclusion, our study indicated that TRIM26 inhibited osteosarcoma progression via promoting proteasomal degradation of RACK1, thereby resulting in inactivation of MEK/ERK signaling, and impeding the EMT process.

## Introduction

Osteosarcoma (OS) is the most common histological subtype of pediatric bone sarcoma that disproportionately affects children and adolescents between the ages of 5 and 20 years, with a second incidence peak in individuals over 50 years of age [1]. Osteosarcoma has a worldwide annual incidence of ~1–3 cases per million [2]. Although it is rare, osteosarcoma is one of the leading causes of cancer-related mortality in children and young adults [3]. With advances in multidisciplinary patient management, neoadjuvant chemotherapy combined with surgical resection is now standard treatment for osteosarcoma patients [4, 5], dramatically increasing the 5-year survival rate from <20% to about 70% in patients with non-metastatic disease [6]. However, for those with metastatic osteosarcoma, most often to the lungs, the 5-year survival rate is ~20% and has remained virtually unchanged over the past 30 years [1, 7]. Lack of understanding of the underlying mechanism in osteosarcoma-genesis had prevented significant improvement in the clinical outcome. Therefore, elucidation of individual osteosarcoma-associated genes regulating its initiation, development, and progression is of great value for identifying potential therapeutic targets and crucial for further osteosarcoma treatment.

The tripartite motif-containing family (TRIMs) proteins belong to the E3 ubiquitin ligase superfamily [8, 9]. Structurally, most TRIM proteins are characterized by a highly conserved tripartite motif at the N-terminus: a RING domain, one or two B-box domains, and a coiled-coil domain [10]. Their C-termini create diversity in TRIM proteins and, on the basis of the differences in their domain structure, TRIM proteins can be stratified into 11 distinct subgroups (C-I–C-XI) [11]. Functionally, TRIMs participate in a broad range of biological processes by acting as E3 ubiquitin ligases including in cellular proliferation, cell cycle progression, DNA damage and repair, ferroptosis, and autophagy[12–18]. Alterations in TRIM expression are associated with diverse pathological conditions, especially cancer [19, 20]. Previous studies have shown that TRIMs play critical roles in oncogenesis and have prognostic value in cancer patients [21–23]. TRIM26, one of the TRIMs family proteins, was involved in the regulation of inflammatory response, cell proliferation, and autophagy. Recently, more attention was paid to the functions of TRIM26 in malignant tumors. For example, TRIM26 was reported to be a tumor suppressor in hepatocellular carcinoma (HCC), and its downregulation was associated with a worse prognosis in HCC patients [24]. Overexpression of TRIM26 inhibited HCC cell proliferation and migration via ubiquitination-dependent degradation of ZEB1 protein and thus impeded the epithelial-to-mesenchymal transition (EMT) process [25]. In addition, enforced expression of TRIM26 in papillary thyroid carcinoma (PTC) impaired cell proliferation and invasion through inactivation of the PI3K/Akt pathway [26]. In another report, TRIM26 exerted an oncogenic role in bladder cancer and depletion of TRIM26 in bladder cancer cells slowed down tumor progress by inhibiting Akt/GSK3β/β-catenin pathway [27]. These previous studies indicated that even the same TRIM family genes may exert opposite functions by targeting different proteins in diverse diseases. However, the specific functional role of TRIM26 in osteosarcoma and its underlying mechanism has not been sufficiently elucidated.

In this study, we found that TRIM26 was downregulated in osteosarcoma, and its expression was an independent protective factor and correlated with a better prognosis in osteosarcoma patients. Enforced expression of TRIM26 significantly inhibited cell proliferation and invasion both in vitro and in vivo. In contrast, the silence of TRIM26 caused the opposite effect. Mechanistically, our results suggested that TRIM26 inhibited osteosarcoma progression via accelerating the degradation of RACK1, and thus resulted in the inactivation of MEK/ERK signaling and impeded the EMT process. This study revealed a novel role of the TRIM26/RACK1 axis in osteosarcoma progression and the underlying mechanism. TRIM26 might be a potential prognostic predictor and a promising therapeutic target for treating osteosarcoma.

## Results

### TRIM26 is downregulated in osteosarcoma samples and cell lines and predicted favorable prognosis in osteosarcoma patients

**Fig. 1:**
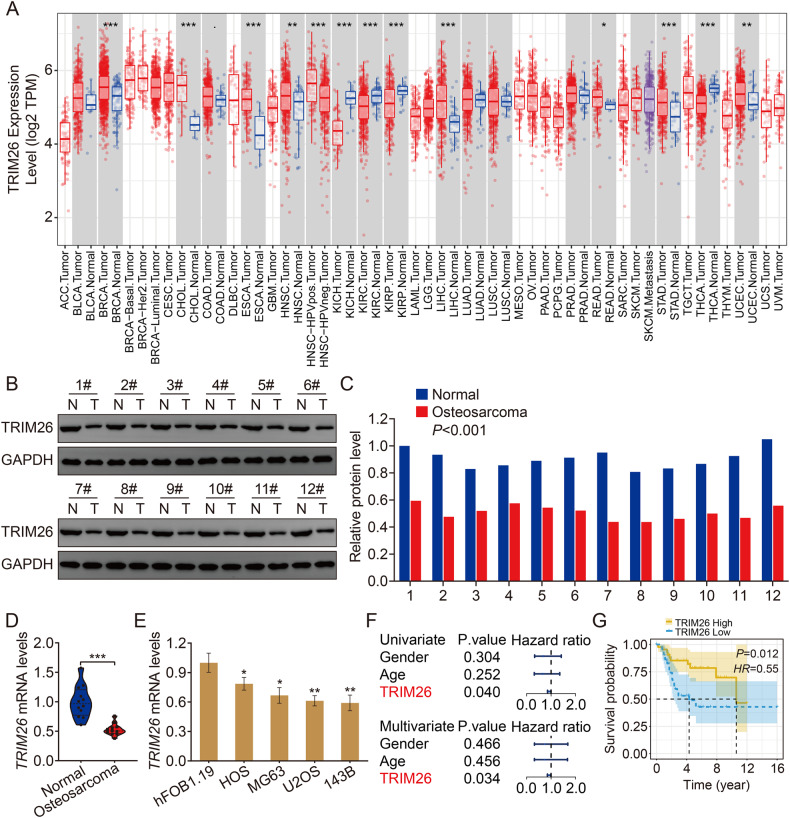
TRIM26 is downregulated in osteosarcoma samples and cell lines and predicted favorable prognosis in osteosarcoma patients. **A** The expression patterns of TRIM26 in cancers and normal tissues from the TCGA dataset based on the TIMER database. **B** Western blot assay was conducted to explore the protein levels of TRIM26 in osteosarcoma tissues and matched normal tissues. **C** Quantitative analysis of the western blot results. **D** The mRNA levels of TRIM26 in osteosarcoma tissues and matched normal tissues were determined by qRT-PCR. **E** The mRNA levels of TRIM26 in osteosarcoma cells (143B, U2OS, MG63, and HOS) and normal osteoblasts (hFOB1.19) were examined by qRT-PCR. **F** Univariate and multivariate Cox regression analyses were performed to evaluate the predictive power of TRIM26 in osteosarcoma based on the TCGA dataset. **G** Kaplan–Meier survival analysis of TRIM26 in osteosarcoma based on TCGA dataset. *P < 0.05, **P < 0.01, *** *P* < 0.001.

To explore the functional role of TRIM26 in osteosarcoma, we first analyzed TRIM26 expression in different tumors represented in the TCGA database. As shown in Fig. 1A, TRIM26 expression varied in different cancer types, with higher expression in breast carcinoma, cholangiocarcinoma, esophageal carcinoma, head and neck squamous cell carcinoma, hepatocellular carcinoma, rectal adenocarcinoma, gastric adenocarcinoma, and endometrial adenocarcinoma compared to adjacent normal tissues and decreased expression in kidney chromophobe, renal cell carcinoma, renal papillary carcinoma, and thyroid carcinoma. Little is known about TRIM26 in osteosarcoma, so we measured its expression in osteosarcoma tissue samples and cell lines. Western blot and qRT-PCR analyses suggested that mRNA and protein levels of TRIM26 were significantly lower in osteosarcoma tissue samples compared to adjacent normal (Fig. 1B–D). Moreover, TRIM26 expression was significantly reduced in osteosarcoma cells (143B, U2OS, MG63, and HOS) compared with normal osteoblasts (hFOB1.19) (Fig. 1E). Univariate and multivariate Cox regression analyses indicated that TRIM26 was a protective factor and an independent prognostic biomarker in osteosarcoma (Fig. 1F). Additionally, Kaplan–Meier survival analysis illustrated that higher expression of TRIM26 was associated with a better prognosis in patients with osteosarcoma (Fig. 1G).

### Overexpression of TRIM26 inhibits osteosarcoma cell proliferation and invasion by inhibiting EMT

**Fig. 2:**
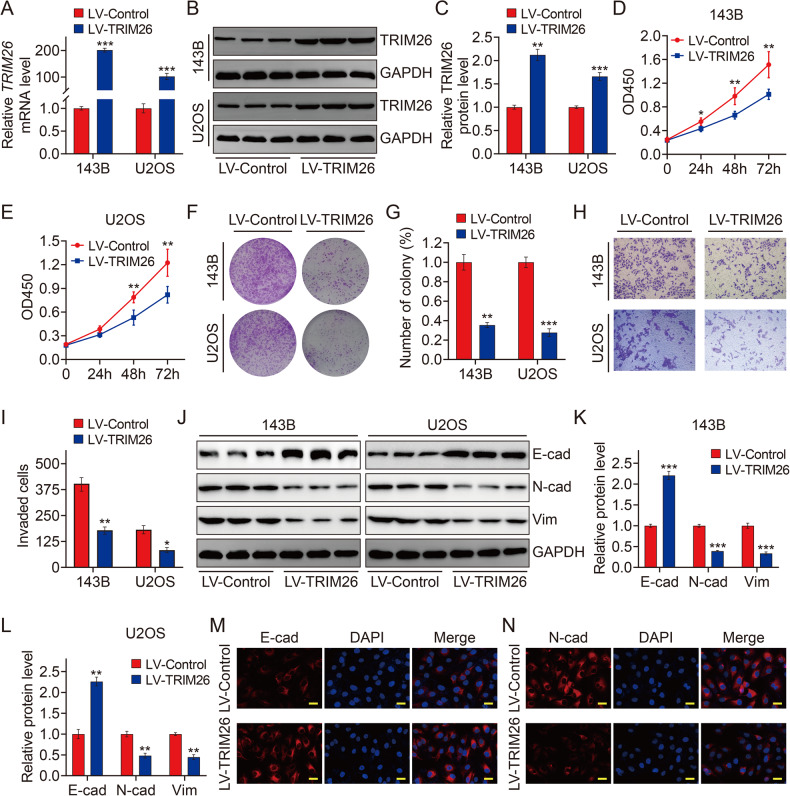
Overexpression of TRIM26 inhibits osteosarcoma cell proliferation and invasion by inhibiting EMT. **A** qRT-PCR analysis of TRIM26 in 143B and U2OS cells after infection of TRIM26 overexpression lentivirus. **B** and **C** Western blot analysis of TRIM26 in osteosarcoma cells after infection of TRIM26 overexpression lentivirus and quantitative analysis. **D** and **E** CCK-8 assays were performed in 143B and U2OS cells after TRIM26 overexpression. **F** and **G** Colony formation assays in 143B and U2OS cells with TRIM26 overexpression, and quantitative analysis. **H** and **I** Transwell invasion assays in 143B and U2OS cells with TRIM26 overexpression, and quantitative analysis. **J** Western blot analysis of EMT-related markers including E-cadherin (E-cad), N-cadherin (N-cad), and vimentin (Vim) in osteosarcoma cells stably overexpressing TRIM26. **K** and **L** Quantitative analysis of protein levels of E-cad, N-cad, and Vim in 143B and U2OS cells with TRIM26 overexpression. **M** and **N** Immunofluorescent staining of E-cad and N-cad in 143B cells stably overexpressing TRIM26 and control cells. Scale bar: 200 μM. *P < 0.05, **P < 0.01, ***P < 0.001.

To gain insights into the role of TRIM26 in osteosarcoma, we upregulated its expression through lentiviral infection. 143B and U2OS cells stably overexpressing TRIM26 was confirmed by qRT-PCR and western blotting analyses (Fig. 2A–C). The CCK-8 and colony formation assays suggested that TRIM26 overexpression inhibited 143B and U2OS cell proliferation (Fig. 2D–G). A transwell invasion assay showed that TRIM26 overexpression reduced cellular invasion (Fig. 2H, I). Given that TRIM26 was reported to be associated with the EMT process, we examined the expression EMT markers including E-cadherin, vimentin, and N-cadherin to investigate the mechanisms underlying a potential TRIM26-driven phenotype. Western blot analysis revealed that overexpression of TRIM26 dramatically inhibited the protein expression of vimentin, and N-cadherin and increased the expression of E-cadherin (Fig. 2J–L). Furthermore, immunofluorescent staining in 143B and U2OS cells also revealed that TRIM26 overexpression reduced the expression intensity of N-cadherin while increasing E-cadherin staining signal (Fig. 2M, N and Supplementary Fig. 1A, B). Taken together, these results suggest that TRIM26 inhibits cell proliferation and invasion by inhibiting EMT in osteosarcoma.

### Silence of TRIM26 accelerates osteosarcoma cell proliferation and invasion through promoting EMT

**Fig. 3:**
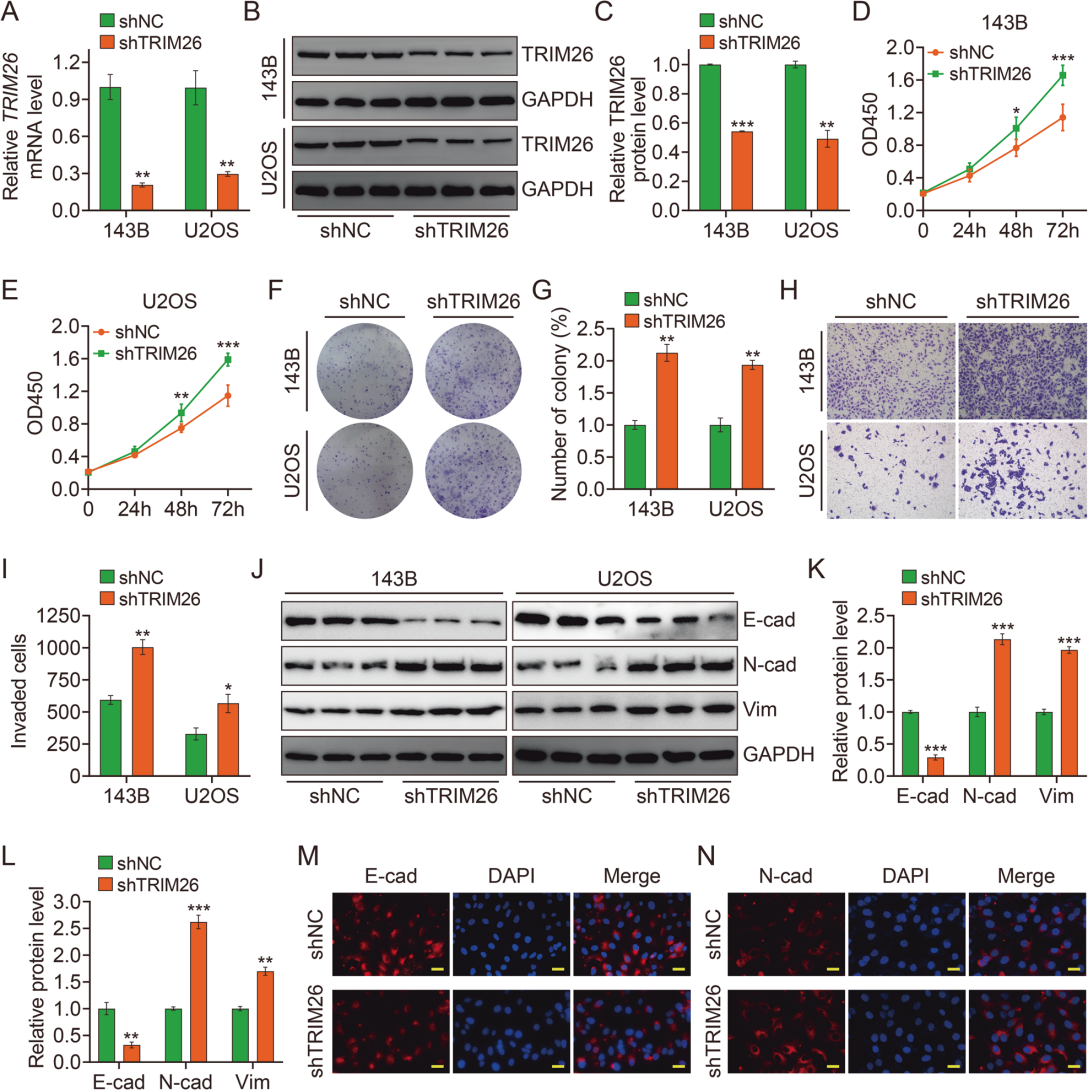
Silence of TRIM26 accelerates osteosarcoma cell proliferation and invasion through promoting EMT. **A** qRT-PCR analysis of TRIM26 in 143B and U2OS cells after infection of shTRIM26 lentivirus. **B** and **C** Western blot analysis of TRIM26 protein levels in osteosarcoma cells after infection of shTRIM26 lentivirus, and quantitative analysis. **D** and **E** CCK-8 assays were performed in 143B and U2OS cells with TRIM26 knockdown. (**F** and **G**) Colony formation assays in 143B and U2OS cells with TRIM26 knockdown, and quantitative analysis. **H** and **I** Transwell invasion assays in 143B and U2OS cells with TRIM26 knockdown, and quantitative analysis. **J** Western blot analysis E-cad, N-cad, and Vim in osteosarcoma cells with TRIM26 knockdown. **K** and **L** Quantitative analysis of protein levels of E-cad, N-cad, and Vim in 143B and U2OS cells with TRIM26 knockdown. **M** and **N** Immunofluorescent staining of E-cad and N-cad in 143B cells stably silencing TRIM26 and control cells. Scale bar: 200 μM. *P < 0.05, **P < 0.01, ***P < 0.001.

To further explore the role of TRIM26 on osteosarcoma cell proliferation and invasion, we silenced its expression by lentivirus-mediated transfection of shTRIM26. The knock-down efficiency was determined by qRT-PCR and western blotting analyses (Fig. 3A–C). The CCK-8 and colony formation assays were further carried out and confirmed that the silence of TRIM26 significantly inhibited cell proliferation in osteosarcoma cells (Fig. 3D–G). The transwell invasion assay revealed that silencing TRIM26 significantly enhanced the invasiveness of osteosarcoma cells (Fig. 3H, I). Furthermore, western blot analysis indicated that TRIM26 knock-down declined E-cadherin expression while increasing the expression of vimentin and N-cadherin (Fig. 3J–L). Additionally, immunofluorescent staining in 143B cells showed that silencing TRIM26 put up the stronger staining signal of N-cadherin while inhibiting the expression intensity of E-cadherin (Fig. 3M, N and Supplementary Fig. 2A, B).

### Overexpression of TRIM26 inhibits MEK/ERK signaling in osteosarcoma

**Fig. 4:**
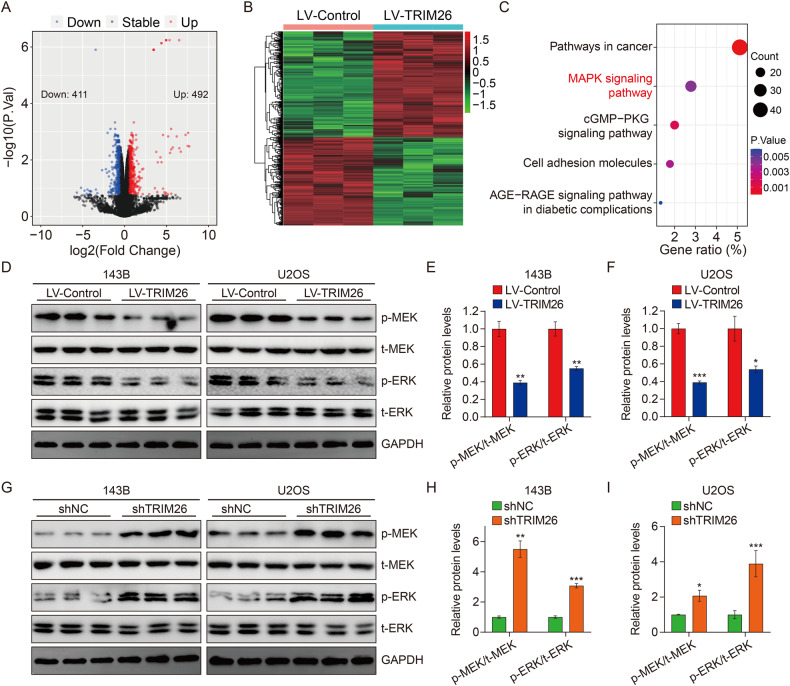
TRIM26 negatively regulates MEK/ERK signaling in osteosarcoma cells. **A** Volcano map of differentially expressed genes between LV-TRIM26 and LV-Control groups. **B** Heatmap exhibits the expression profiles of the DEGs. **C** KEGG pathway enrichment analysis of the DEGs. **D**–**F** Western blot analysis of MEK1/2, p-MEK1/2, ERK1/2, and p-ERK1/2 protein levels in osteosarcoma cells with TRIM26 overexpression and quantitative analysis. **G**–**I** Western blot analysis of MEK1/2, p-MEK1/2, ERK1/2, and p-ERK1/2 protein levels in osteosarcoma cells with TRIM26 knockdown, and quantitative analysis. *P < 0.05, **P < 0.01, ***P < 0.001.

To explore the potential molecular mechanism underpinning TRIM26-mediated inhibition of osteosarcoma cell proliferation and invasion, we performed RNA-Seq after TRIM26 overexpression in biological duplicates in 143B cells. A total of 903 differentially expressed genes (DEGs) were identified, with 492 or 411 genes being upregulated or downregulated after TRIM26 overexpression (Fig. 4A, B). KEGG-enrichment analysis of these DEGs revealed that the MAPK signaling pathway was significantly correlated with TRIM26 expression (Fig. 4C). MEK/ERK signaling is one of the typical MAPK signaling that plays vital roles in tumor cell proliferation, migration, and invasion, so we examined the effect of TRIM26 on MEK/ERK activation in osteosarcoma cells by examining the expression of MEK1/2, p-MEK1/2, ERK1/2, and p-ERK1/2 by western blotting. TRIM26 overexpression decreased protein expression of p-MEK1/2 and p-ERK1/2 (Fig. 4D–F) and, conversely, TRIM26 knockdown increased the phosphorylation of MEK1/2 and ERK1/2 (Fig. 4G–I). These results suggest that TRIM26 negatively regulates MEK/ERK signaling in osteosarcoma cells.

### TRIM26 binds to and degrades RACK1

**Fig. 5:**
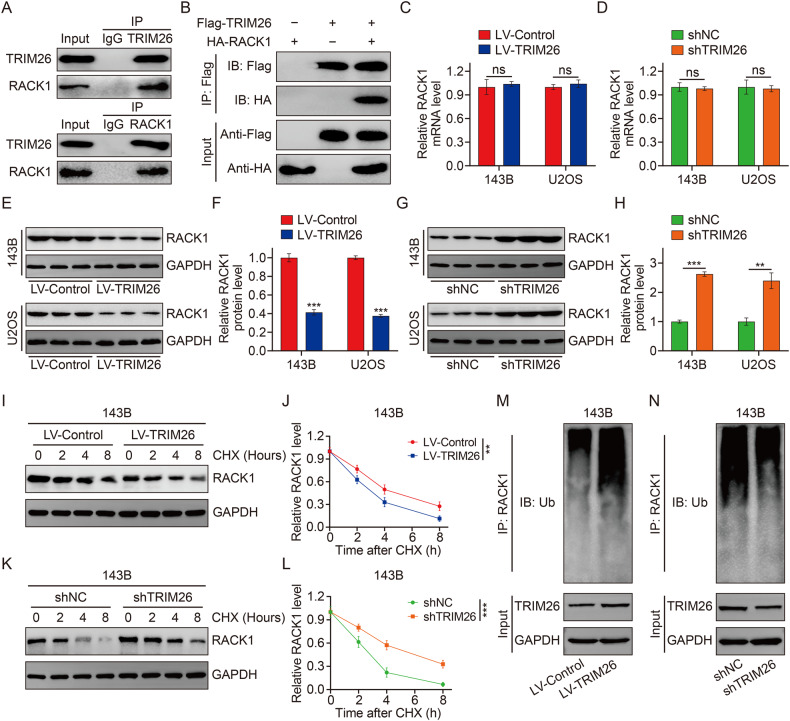
TRIM26 binds to and degrades RACK1. **A** Endogenous protein interaction between RACK1 and TRIM26 in 143B cell lysates. **B** Exogenous protein interactions between RACK1 and TRIM26 in HEK 293T cells transfected with Flag-TRIM26 and HA-RACK1 plasmid. **C** and **D** qRT-PCR analysis of RACK1 mRNA levels in osteosarcoma cells. **E** and **F** Western blot analysis of RACK1 protein levels in osteosarcoma cells stably overexpressing TRIM26 and quantitative analysis. **G** and **H** Western blot analysis of RACK1 protein levels in TRIM26-silenced osteosarcoma cells and quantitative analysis. **I** and **J** Western blot analysis of RACK1 protein levels in TRIM26-upregulated cells and corresponding control cells in the presence of CHX (10 μg/ml) for indicated time point. **K** and **L** Western blot analysis of RACK1 protein levels in TRIM26-silenced cells and corresponding control cells in the presence of CHX (10 μg/ml) for indicated time point. **M** Evaluation of endogenous RACK1 ubiquitination in 143B cells transfected with LV-Control or LV-TRIM26. **N** Evaluation of endogenous RACK1 ubiquitination in 143B cells transfected with shNC or shTRIM26. **P < 0.01, *** *P* < 0.001.

To further investigate the underlying mechanism by which TRIM26 regulates MEK/ERK signaling in osteosarcoma, TRIM26-interacting proteins were identified according to previous research [16]. The receptor for activated C kinase 1 (RACK1), a classic scaffolding protein that participates in the protein kinase C (PKC) signaling pathway, was identified as a protein that might interact with TRIM26. Cellular immunofluorescence experiments to confirmed the co-localization of RACK1 and TRIM26 in 143B and U2OS cells (Supplementary Fig. 3). Immunoprecipitation assay in 143B cells suggested that RACK1 was precipitated by TRIM26 and reverse immunoprecipitation confirmed that TRIM26 could also be precipitated by RACK1 in osteosarcoma cells (Fig. 5A). Moreover, in HEK 293T cells, Co-IP assay showed that Flag-tagged TRIM26 coprecipitated with HA-tagged RACK1 and HA-tagged RACK1 coprecipitated with Flag-tagged TRIM26 efficiently (Fig. 5B and Supplementary Fig. 4). Taken together, the results we have presented so far indicated that TRIM26 could interact with RACK1. Then, we explored the influence of silencing and overexpressing TRIM26 on RACK1 expression in osteosarcoma cells. qRT-PCR results indicated that TRIM26 knockdown or overexpression had no influence on RACK1 mRNA expression level (Fig. 5C, D), but resulted in increased or decreased RACK1 protein levels (Fig. 5E–H). Thus, we can speculate that TRIM26 regulates RACK1 at the protein level but not at the mRNA level. Further, we explored the effect of TRIM26 on the protein stability of endogenous RACK1 in the presence of protein synthesis inhibitor cycloheximide (CHX). As shown in Fig. 5I–L, the silence of TRIM26 significantly inhibited the degradation of RACK1, whereas overexpression of TRIM26 markedly promoted RACK1 degradation. Considering that TRIM26 could function as an E3 ubiquitination ligase, we further explored the effect of TRIM26 knockdown or overexpression on the ubiquitination of RACK1. As shown in Fig. 5M, N, we found that overexpression of TRIM26 increased the ubiquitination of RACK1, whereas depletion of TRIM26 significantly decreased RACK1 ubiquitination in osteosarcoma cells. Collectively, these findings indicated that TRIM26 could interact with RACK1 and promote the degradation of RACK1 through ubiquitination.

### TRIM26 inhibits osteosarcoma cell proliferation and invasion though RACK1-mediated inactivation on MEK/ERK signaling

**Fig. 6:**
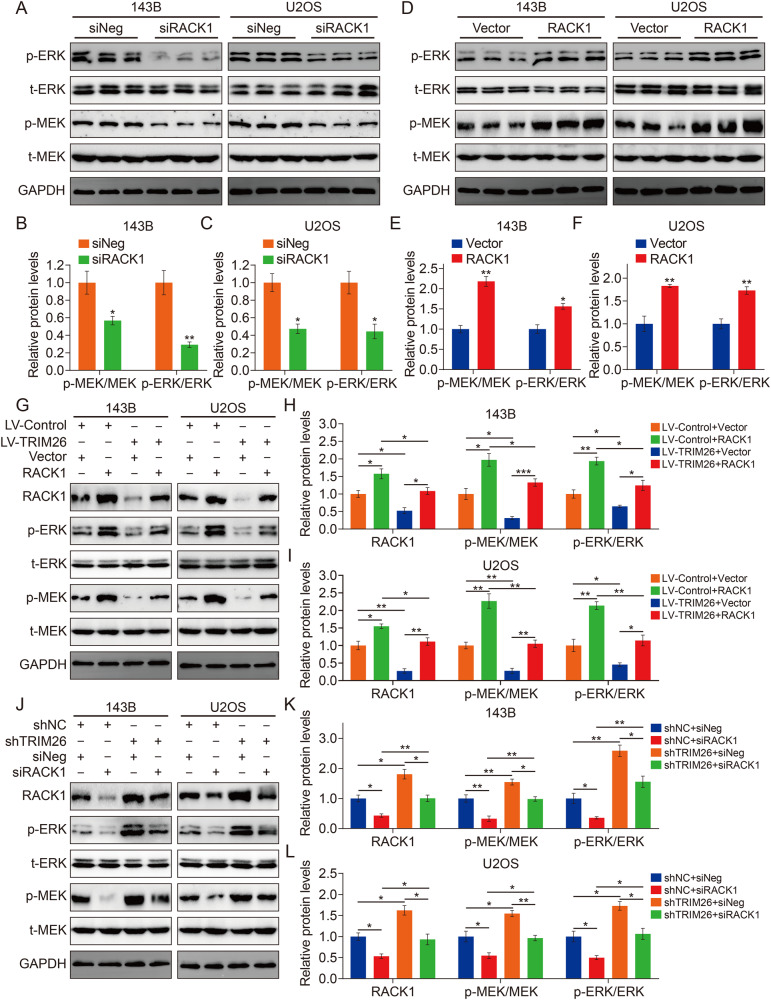
RACK1-mediated the effect of TRIM26 on MEK/ERK signaling pathway. **A**–**C** Western blot analysis of MEK1/2, p-MEK1/2, ERK1/2, and p-ERK1/2 protein levels in osteosarcoma cells after silencing RACK1, and quantitative analysis. **D**–**F** Western blot analysis of MEK1/2, p-MEK1/2, ERK1/2, and p-ERK1/2 protein levels in osteosarcoma cells after overexpressing RACK1 and quantitative analysis **G**–**I** Evaluation of the phosphorylation of MEK1/2 and ERK1/2 in TRIM26-upregulated cells after transfection of RACK1 overexpression plasmid and empty vector. **J**–**L** Evaluation of the phosphorylation of MEK1/2 and ERK1/2 in TRIM26-silenced cells after transfection of siRACK1 and siNeg. *P < 0.05, **P < 0.01, ***P < 0.001.

We further investigated the role of RACK1 in the mechanism by which TRIM26 inhibits the progression of osteosarcoma. First, we detected the effect of RACK1 knockdown or overexpression on the activation of MEK/ERK signaling. As shown in Fig. 6A–C, the silence of RACK1 decreased the phosphorylation of MEK1/2 and ERK1/2. Conversely, overexpression of RACK1 increased the expression levels of p-MEK1/2 and p-ERK1/2 (Fig. 6D–F). Moreover, we manipulated the expression of RACK1 in TRIM26-upregulated or TRIM26-silenced osteosarcoma cells through plasmid or siRNA transfection. Western blot results indicated that RACK1 overexpression could partly attenuate the inhibitory effect of TRIM26 overexpression on p-MEK1/2 and p-ERK1/2 (Fig. 6G–I), and silence of RACK1 impaired the effect of TRIM26 knockdown-induced upregulation of p-MEK1/2 and p-ERK1/2 (Fig. 6J–L). Several in vitro functional rescue experiments were performed in TRIM26-upregulated or TRIM26-silenced osteosarcoma cells after overexpressing or silencing RACK1, and the results showed that the decreased malignant behaviors including cell proliferation and invasion in TRIM26-upregulated cells were reversed when RACK1 was overexpressed, whereas RACK1 knockdown diminished the increased malignant phenotypes in TRIM26-silenced osteosarcoma cells (Supplementary Fig. 5A–F).

### TRIM26 inhibits tumor growth in vivo

**Fig. 7:**
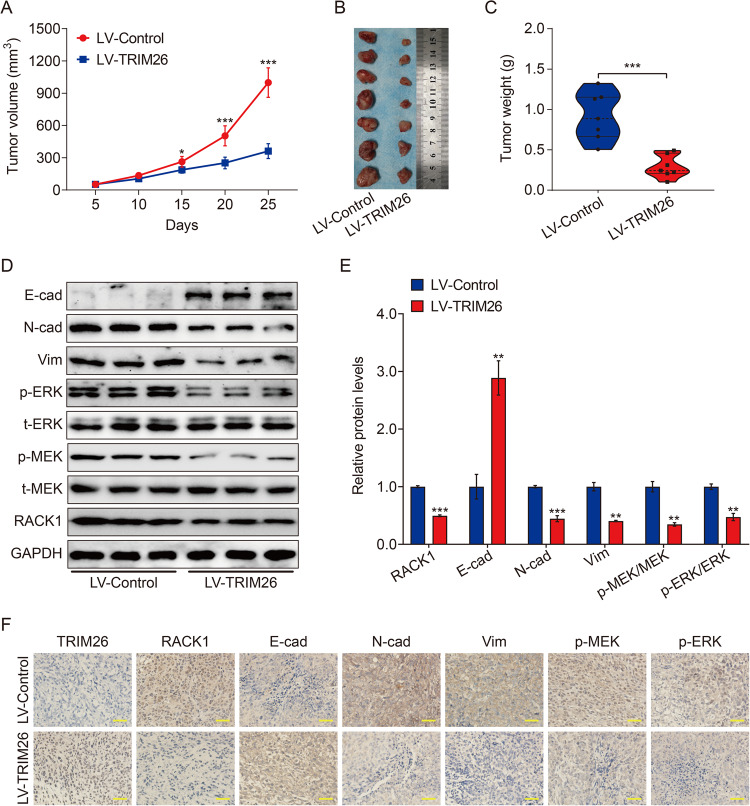
TRIM26 inhibits tumor growth in vivo. **A** The growth curve of the xenografts in LV-Control and LV-TRIM26 groups. **B** Illustration of tumors excised from nude mice in LV-Control and LV-TRIM26 groups. **C** Comparison of the excised tumor weight between the LV-Control and LV-TRIM26 groups. **D** and **E** Western blot analysis of RACK1, E-cad, N-cad, Vim, MEK1/2, p-MEK1/2, ERK1/2, and p-ERK1/2 protein in xenografts tumors, and quantitative analysis. **F** Immunohistochemical analysis of RACK1, E-cad, N-cad, Vim, p-MEK1/2, and p-ERK1/2 in tumor xenografts. Scale bar: 200 μM. *P < 0.05, **P < 0.01, ***P < 0.001.

Finally, we tested whether TRIM26 can exert similar tumor-inhibitory activity in vivo by using a xenografts mouse model. 143B cells bearing LV-TRIM26 or LV-Control were subcutaneously injected into the nude mice (seven mice per group, *n* = 7), and xenograft volumes and weights were monitored. As shown in Fig. 7A, tumor growth curve results indicated that TRIM26 overexpressed xenografts, as confirmed by western blotting and immunohistochemistry, grew significantly slower than control xenografts. The xenograft tumors were removed 25 days after the injection of 143B cells. As shown in Fig. 7B, C, TRIM26-overexpressed 143B xenografts were significantly smaller and lighter than control xenografts expressing LV-Control. Moreover, analysis of RACK1, MEK/ERK signaling, and EMT-related markers in subcutaneous tumors by western blotting and immunohistochemistry indicated that RACK1, p-MEK1/2, p-ERK1/2, vimentin, and N-cadherin were significantly lower, and the expression of E-cadherin was significantly higher in the LV-TRIM26 group than those in the LV-Control group (Fig. 7D–F). Taken together, these results suggest that overexpression of TRIM26 inhibits tumor growth by destabilizing RACK1 and thus inactivation of MEK/ERK signaling in osteosarcoma.

## Discussion

There is an increasing body of evidence suggesting that TRIM family proteins play critical roles in various physiological processes and their dysregulations are involved in carcinogenesis and cancer progression [9, 10, 28]. Members of the TRIM family participate in tumor cell proliferation, metastasis, and chemoresistance in both E3 ubiquitin ligase-dependent or -independent manners [29–31]. Many TRIMs have also been found to be aberrantly expressed in osteosarcoma and could function as tumor suppressor genes or oncogenes. For instance, TRIM22 was found to inhibit osteosarcoma progression via destabilizing NRF2, which subsequently resulted in the imbalance of intracellular reactive oxygen species (ROS) and further activation of AMPK/mTOR/autophagy signaling [32]. TRIM17 was shown to play a vital role in regulating tumorigenesis and chemoresistance in osteosarcoma via ubiquitination of breast cancer metastasis suppressor 1 (BRMS1). Furthermore, TRIMs have also been reported as prognostic in osteosarcoma [33].

In this study, we found that TRIM26 was a protective factor in osteosarcoma and that its lower expression predicted a worse clinical outcome, indicating a tumor-suppressive role for TRIM26 in osteosarcoma. Moreover, analyzing its expression in osteosarcoma cells and tumor tissues suggested that TRIM26 expression was dramatically lower in osteosarcoma tissues and cell lines compared with normal. Subsequently, we manipulated TRIM26 expression in osteosarcoma cells and performed functional experiments. Our results suggested that TRIM26 overexpression dramatically inhibited the proliferation of osteosarcoma cells, and reduced cellular invasion. In contrast, the silence of TRIM26 caused the opposite effect. In addition, overexpression of TRIM26 markedly inhibited tumor growth in the xenografts mouse model, further supporting the tumor-suppressive role of TRIM26 in osteosarcoma. Collectively, these results suggest that TRIM26 was a potential prognostic predictor and a promising therapeutic target for treating osteosarcoma.

To explore the underlying mechanism by which TRIM26 inhibits osteosarcoma progression, we performed RNA-seq analysis and found that TRIM26 overexpression-induced-differentially expressed genes were particularly enriched in mitogen-activated protein kinase (MAPK) signaling pathway according to the KEGG analysis. Therefore, we speculated that TIRM26 might inhibit the malignant behaviors in osteosarcoma by regulating the MAPK cascade. The MAPK signaling pathway is a highly conserved intracellular pathway that consists of three hierarchically sequential kinase components: MAPK kinase-kinase (MAPKKKs), MAPK kinase (MAPKKs), and MAPKs. Most upstream kinases (MAPKKKs) are activated by various extra- and intracellular stimuli and subsequently phosphorylate the middle kinase (MAPKKs), which in turn exclusively phosphorylate and activate MAPKs [34–36]. In mammals, MAPKs include extracellular signal-regulated kinase (ERK), c-Jun NH2-terminal kinase (JNK), and p38 MAPK [37]. These MAPKs have a large spectrum of substrates that execute specific cell fate decisions in a highly context-dependent manner [38]. The canonical MAPK/ERK pathway attracted much attention since it was one of the most important signaling cascades among all MAPK signaling pathways and was essential for tumor cell survival, dissemination, and resistance to chemotherapy [39, 40]. Recent research revealed that the MAPK/ERK signaling could serve as a tumor suppressor and more commonly function as pro-oncogenic signaling, which was associated with the timing, duration, and intensity of its signal [41–43]. To investigate whether the MAPK/ERK signaling act downstream of TRIM26, we examined the phosphorylation of ERK1/2 and its kinase (MEK1/2) in osteosarcoma cells after TRIM26 overexpression and knockdown, and our results showed that TRIM26 negatively regulates MEK/ERK signaling cascade in osteosarcoma cells. Thus, we could conclude that TRIM26 inhibited osteosarcoma proliferation and invasion via inactivation of MEK/ERK signaling.

Further mechanistic studies of the TRIM26-induced cancer-inhibiting effects revealed that TRIM26 regulates MEK/ERK signaling by promoting the degradation of RACK1. RACK1, a highly conserved tryptophan-aspartate repeat protein, has a seven-bladed propeller that enables it to be a scaffold protein for multiple kinases and receptors [44]. RACK1 plays a critical role in multiple intracellular processes, including protein transporting, protein stability, and protein activity [45]. Aberrant expression of RACK1 has been observed in several kinds of cancers, and it was demonstrated to exert either suppressive or more commonly promotive effects on cancers [46, 47]. For instance, upregulation of RACK1 expression accelerated tumorigenesis, progression, and metastasis in multiple tumors including hepatocellular carcinogenesis, oral squamous cell carcinoma [48], cervical cancer [49], and colon cancer [50], while it was reported to be a tumor suppressor gene in gastric cancer [51]. However, it is still unclear about the role of RACK1 in osteosarcoma. In this study, it was demonstrated that TRIM26 interacted with RACK1 and promoted its degradation in a ubiquitination-dependent manner. Rescue experiments revealed that RACK1 mediated the effect of TRIM26-induced inhibition of MEK/ERK signaling. In addition, in vitro functional assays indicated that overexpression of RACK1 could reverse the decreased malignant behaviors in TRIM26-upregulated cells. Therefore, we could conclude that TRIM26 inhibits cell proliferation and invasion in osteosarcoma through destabilizing RACK1 and thus inactivation of MEK/ERK signaling. RACK1 was reported to participate in the protein kinase C (PKC) signaling pathway by shutting active PKCs to their substrates and PKCs could serve as the upstream of MEK/ERK signaling [52, 53]. Therefore, the possible mechanism for which RACK1 knockdown-induced inactivation of MEK/ERK signaling was via PKCs and needs further exploration.

In conclusion, our study provided evidence of the tumor-suppressive role of TRIM26 in osteosarcoma. Lower expression of TRIM26 was associated with worse overall survival in patients with osteosarcoma. TRIM26 inhibited osteosarcoma progression via promoting the degradation of RACK1, and thus inactivation of the MEK/ERK pathway. In the future, TRIM26 might be served as a candidate prognostic biomarker and a potential target for new therapies in osteosarcoma.

## Material and methods

### Cell culture and lentivirus infection

The human osteosarcoma cell lines U2OS, MG63, and HOS were obtained from the Cell Bank of Wuhan University (Wuhan, China) and Cell Bank of Type Culture Collection (CBTCC, Chinese Academy of Sciences, Shanghai, China). The human osteoblast cell line hFOB1.19 and osteosarcoma cell line 143B were purchased from Servicebio Technology (Wuhan, China). Cells were cultured in a complete medium supplemented with 10% FBS (Gibco, Thermo Fisher Scientific, Waltham, MA, USA) and 1% antibiotics (100 units/ml penicillin and 100 units/ml streptomycin) in a humidified incubator at 37 °C and 5% CO_2_ atmosphere. Lentivirus containing pLVX-TRIM26-Puro, pLVX-shTRIM26-Puro (5’-GCCTGTACAAGAGTGCCTA-3’), and corresponding control lentivirus were purchased from Obio Technology (Shanghai, China) and were used to infect 143B and U2OS cells according to the manufacturer’s protocol. Infected cells were then subjected to puromycin selection (5 μg/ml), and stable transfection of cells was confirmed by western blotting.

### Tissue collection

Twelve paired tissue samples, including 12 osteosarcoma tissues and 12 adjacent normal tissues, were collected from patients with a histopathological diagnosis of osteosarcoma undergoing surgery at Renmin Hospital of Wuhan University between July 2020 and June 2022. All patients signed informed consent, and the research and the Research Ethics Committee of Renmin Hospital of Wuhan University approved the study protocol.

### Cell proliferation and transwell invasion assays

Osteosarcoma cells were trypsinized and resuspended in a complete medium. For the CCK-8 assay, cells were cultured in 96-well plates at an initial density of 3 × 10^3^ cells/well. CCK-8 reagent (Servicebio Technology) was added into each well at 0, 24, 48, and 72 h. After incubation for 1 h at 37 °C, the absorbance readings were obtained at 450 nm. For the colony formation assay, cells were seeded into six-well plates at an initial density of 5 × 10^2^ cells/well and were cultured for ~10 days. Colonies were fixed with 4% paraformaldehyde and stained with 0.1% crystal violet. For the transwell invasion assay, 1 × 10^5^ cells in 200 μl serum-free medium were added into the upper well of a transwell chamber (Corning Costar, Corning, NY, USA) precoated with Matrigel, and 600 μl medium containing 20% FBS was added into the lower chamber. Invaded cells were fixed with 4% paraformaldehyde and stained with 0.1% crystal violet and observed by inverted microscopy (Olympus, Tokyo, Japan).

### RNA isolation, RNA-Sequence, and qRT-PCR

Briefly, total RNA was extracted using TRIzol reagent (Invitrogen, Carlsbad, CA, USA) according to the manufacturer’s guidelines. 1 μg of total RNA was utilized to synthesize cDNA using the RevertAid First Strand cDNA Synthesis Kit (Thermo Fisher Scientific, Waltham, MA, USA). For RNA-sequence analysis, after being quantified and qualified using Agilent 2100 (Agilent Technologies, Santa Clara, CA, USA), isolated RNA was sent to BGI (BGI Group, Shenzhen, China) for RNA-seq analysis as previously described [54]. Quantitative real-time PCR was performed using SYBR Green Master Mix (BD Biosciences, East Rutherford, NJ, USA). The primers of target genes were obtained from Servicebio Technology and the sequences were: TRIM26, forward, 5’-ACCCATTGCTCGAGTGGTTA-3’ and reverse, 5’-ACTTCCCAGTAGACCTTGCC-3’; RACK1, forward, 5’- GTATTTGCACACACCCAGGG-3’ and reverse, 5’-CAACCAGGTCAGCGATGAAG-3’; GAPDH, forward, 5’-CTGAGTACGTCGTGGAGTCC-3’ and reverse, 5’-GTCTTCTGGGTGGCAGTGAT-3’. The relative expression levels of target genes were calculated using the comparative CT (ΔΔCT) method.

### Western blotting

Proteins were extracted from tissue samples and cells using RIPA lysis buffer in the presence of protease and phosphatase inhibitors. After determining the protein concentration using the BCA method, 20 μg of protein was separated on 10% SDS–PAGE gel and then transferred onto PVDF membranes. After blocking with 5% non-fat milk and washing with TBST three times, the membranes were incubated with specific antibodies overnight at 4 °C. The next day, membranes were incubated with horseradish peroxidase-conjugated secondary antibodies (Beyotime, Nantong, China) for 1 h at room temperature. The ECL kit (Servicebio Technology) was used to visualize membranes. A primary antibody targeting TRIM26 (sc-393832, 1:500) was obtained from Santa Cruz Biotechnology (Dallas, TX, USA), while primary antibodies targeting RACK1 (#5432, 1:500), MEK1/2(#8727, 1:500), p-MEK1/2(#2338, 1:500), ERK1/2 (#4695, 1:500), p-ERK1/2 (#8544, 1:1000), E-cadherin (#3195, 1:1000), N-cadherin (#13116, 1:500), and vimentin (#5741, 1:500) were purchased from Cell Signaling Technology (Danvers, MA, USA).

### Immunoprecipitation (IP)

143B cells and HEK 293T cells were collected and lysed with IP buffer [50 mM Tris–HCl, 150 mM NaCl, 1 mM ethylenediaminetetraacetic acid (EDTA), 1% Triton X-100] containing protease inhibitors (Beyotime, China). After being precleared with protein A/G-agarose beads (Cell Signaling Technology, USA) at 4 °C for 1 h, cell lysates were immunoprecipitated with the indicated antibodies at 4 °C overnight with gentle shaking. After that, the lysates were incubated with protein A/G-agarose beads for 2 h and the nuclear pellets were collected following centrifugation at 3000 rpm at 4 °C. After being washed three times with IP buffer. The bound proteins were then eluted by boiling at 95 °C for 5 min and detected by western blot analysis.

### In vivo ubiquitination assays

Osteosarcoma cells were collected and lysed with IP buffer protease inhibitors (Beyotime, China). Anti-RACK1 antibodies were used to immune-precipitate the endogenous RACK1 at 4 °C overnight with gentle shaking. Then, cell lysates were incubated with protein A/G-agarose beads for another 2 h to form the immunocomplex precipitation. After centrifugation at 3000 rpm at 4 °C, the supernatants were removed and the agarose beads were washed with IP buffer to remove unbound proteins. The pulled-down proteins were subjected to immunoblotting and signals were detected using the ubiquitin antibodies (Abcam, UK).

### In vivo experiments

Four-week-old male BALB/c nude mice were purchased from the Beijing HFK Experiment Animal Center (Beijing, China) and housed in the Animal Center of Renmin Hospital of Wuhan University. After adapting to the environment, 5 × 10^6^ 143B cells with control or TRIM26 overexpression were implanted into nude mice. Tumor volumes were monitored every 5 days as previously described. Twenty-five days after injection, all mice were sacrificed, and the tumors were excised and weighed. The care of the laboratory animals and animal experiments were performed in accordance with the animal ethics guidelines and approved protocols of Renmin Hospital of Wuhan University.

### Immunohistochemistry

Briefly, formalin-fixed, paraffin-embedded tissue sections were dewaxed with xylene. After rehydration and antigen retrieval, sections were blocked with 5% BSA for 60 min and then incubated with the primary antibodies at 4 °C overnight. The antibodies included: anti-TRIM26 antibody (1:200, Santa Cruz Biotechnology), anti-RACK1 (1:200, Cell Signaling Technology), anti-p-MEK1/2 (1:200, Cell Signaling Technology), anti-p-ERK1/2 (1:200, Cell Signaling Technology), anti-E-cadherin (1:200, Cell Signaling Technology), anti-N-cadherin (1:200, Cell Signaling Technology), and anti-vimentin (1:200, Cell Signaling Technology). The next day, sections were stained with diaminobenzidine followed by counterstaining with hematoxylin and washing in water. The sections were observed and photographed under a ×200 objective by light microscopy (Olympus, Japan).

### Statistical analysis

R 4.1.0 software and GraphPad Prism 8 software (GraphPad Software, La Jolla, CA, USA) was used for statistical analysis. All the experiments were conducted in at least three biological duplicates. Differences between groups were compared using unpaired two-tailed Student’s *t*-tests and one-way analysis of variance (ANOVA). A *P*-value < 0.05 was regarded as statistically significant.

## Supplementary information

aj-checklist

Supplementary Figures

Original Data File

## Data Availability

The published article includes all data sets generated/analyzed for this study.
