# Supplementary material for: TRIM26 inhibited osteosarcoma progression through destabilizing RACK1 and thus inactivation of MEK/ERK signaling
Source: Cell Death Dis. 2023 Aug 17;14(8):529. doi: 10.1038/s41419-023-06048-9 (PMC10435491; doi:10.1038/s41419-023-06048-9)
Supplement: Supplementary file 2 — Supplementary Figures [file 41419_2023_6048_MOESM2_ESM.docx]

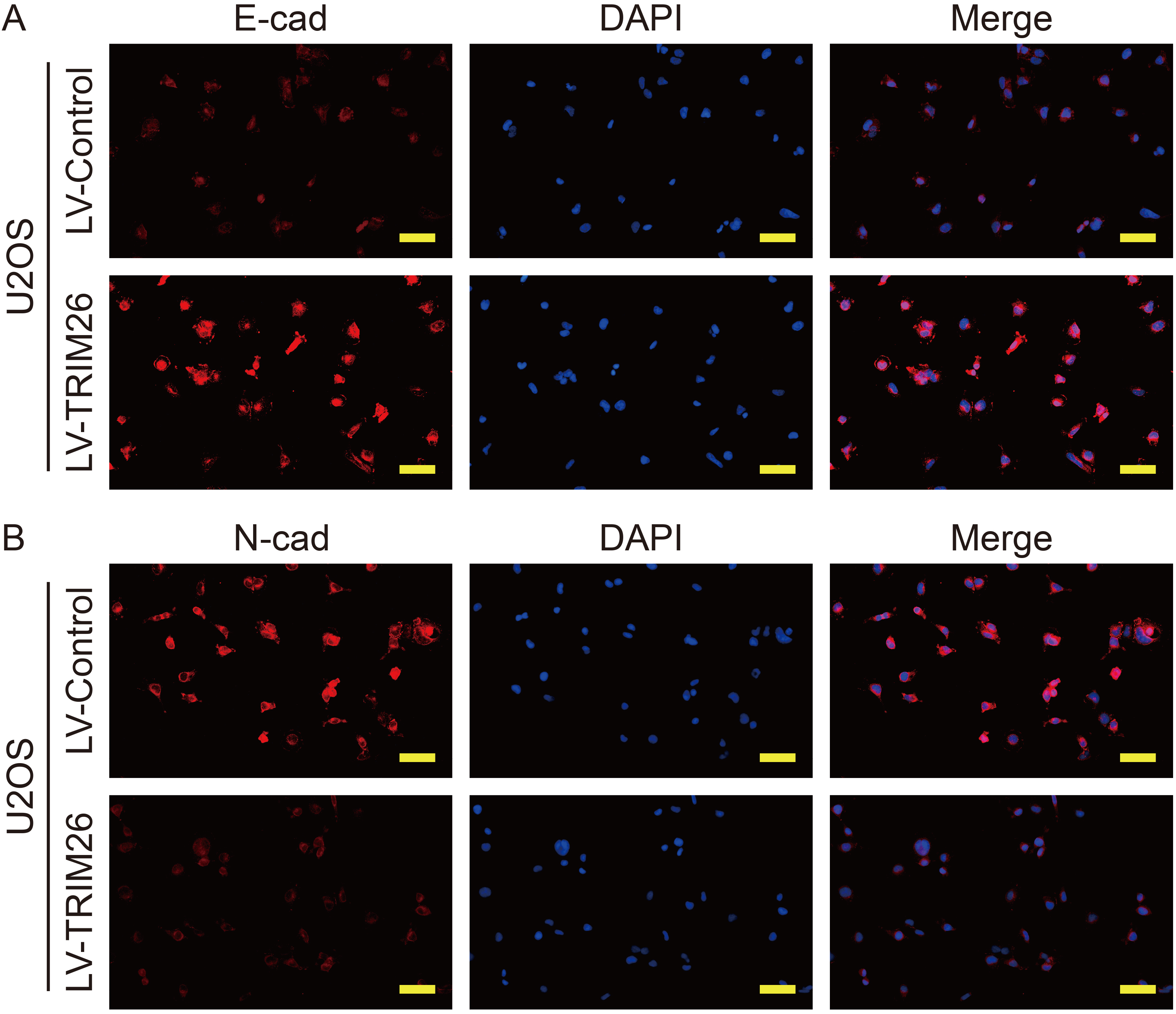


**Supplementary Figure 1.** Immunofluorescent staining of E-cad (A) and N-cad (B) in U2OS cells stably overexpressing TRIM26 and control cells. Scale bar: 200 μM.


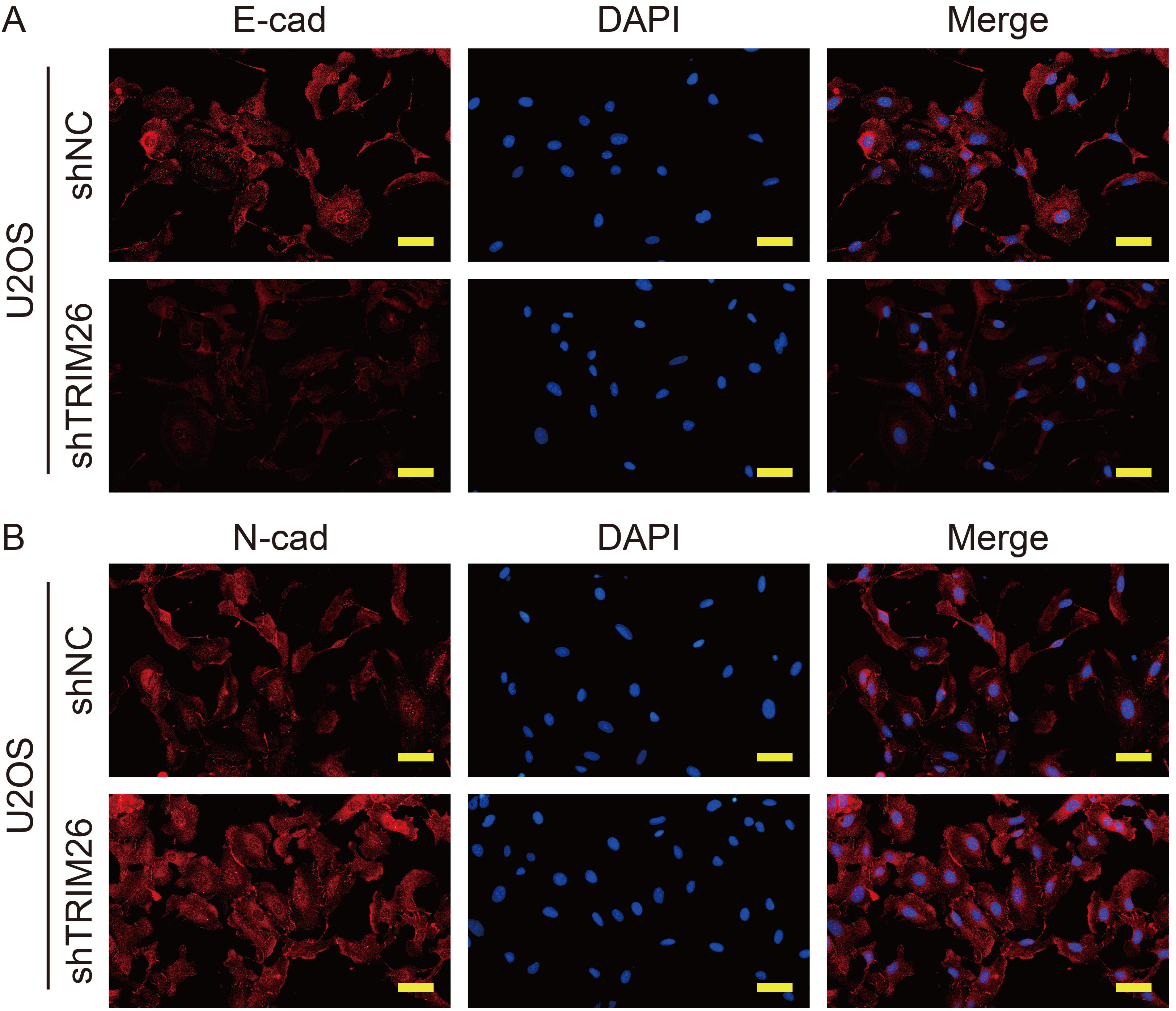


**Supplementary Figure 2.** Immunofluorescent staining of E-cad (A) and N-cad (B) in U2OS cells stably silencing TRIM26 and control cells. Scale bar: 200 μM.


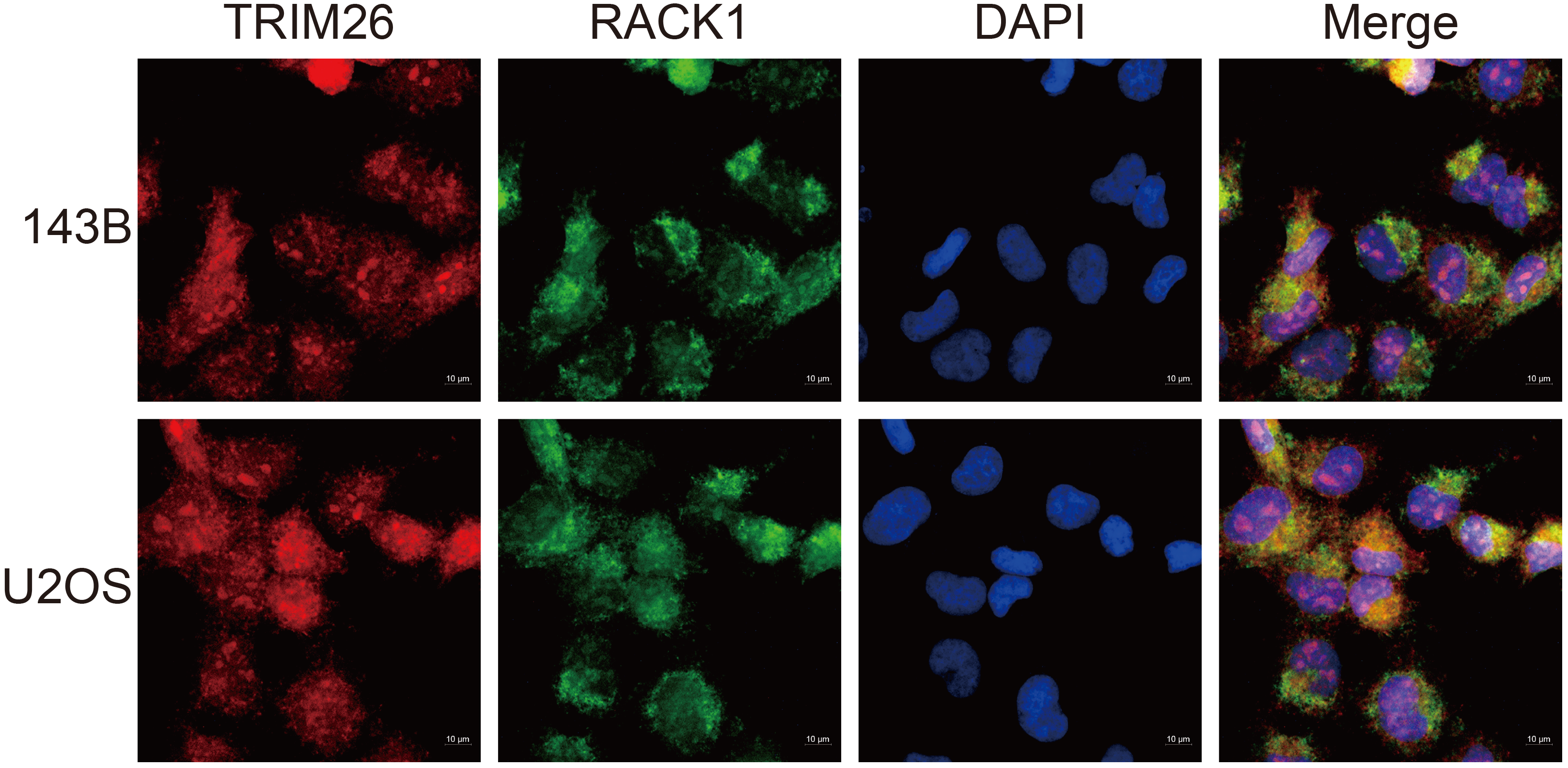


**Supplementary Figure 3.** Co-localization of RACK1 and TRIM26 in 143B and U2OS cells.


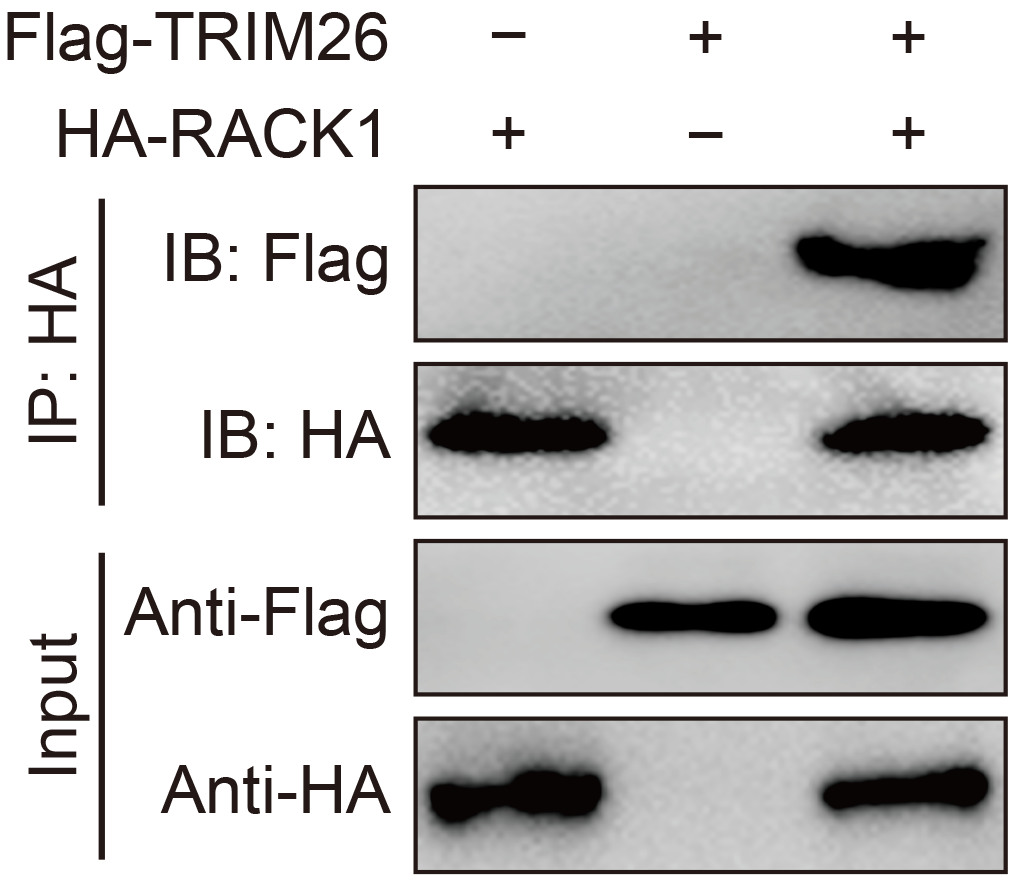


**Supplementary Figure 4.** Exogenous protein interactions between RACK1 and TRIM26 in HEK 293T cells transfected with Flag-TRIM26 and HA-RACK1 plasmid.


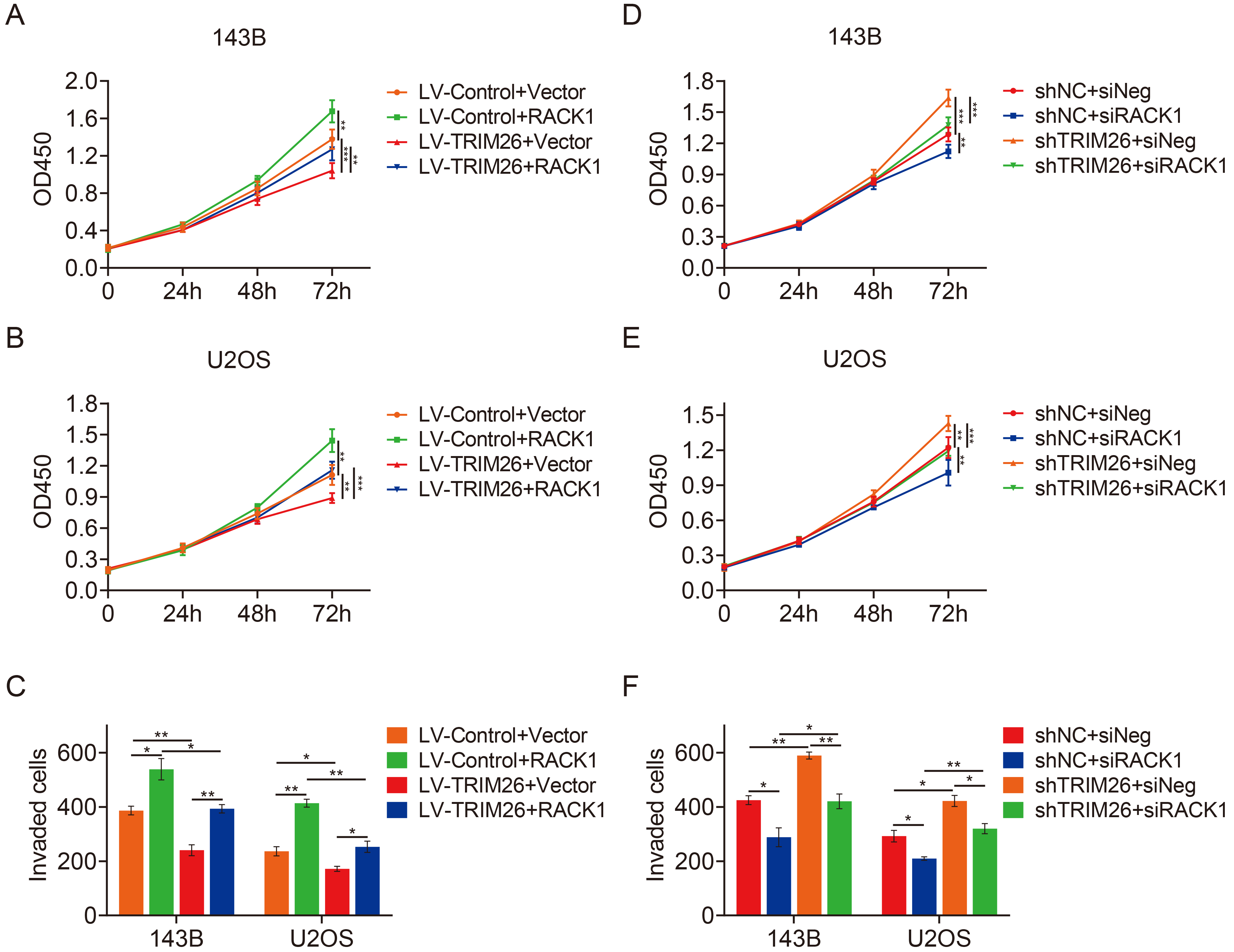


**Supplementary Figure 5. RACK1 mediates the effect of TRIM26 on malignant behavior of osteosarcoma cells.** (A-C) Cell proliferation and invasion abilities were assessed by CCK-8 and transwell invasion assays in TRIM26-upregulated cells after overexpression of RACK1. (D-F) Cell proliferation and invasion abilities were assessed in TRIM26-silenced cells after knockdown of RACK1.
