# Supplementary material for: TRIM26 inhibited osteosarcoma progression through destabilizing RACK1 and thus inactivation of MEK/ERK signaling
Source: Cell Death Dis. 2023 Aug 17;14(8):529. doi: 10.1038/s41419-023-06048-9 (PMC10435491; doi:10.1038/s41419-023-06048-9)
Supplement: Supplementary file 3 — Original Data File [file 41419_2023_6048_MOESM3_ESM.pdf]

Fig. 1B

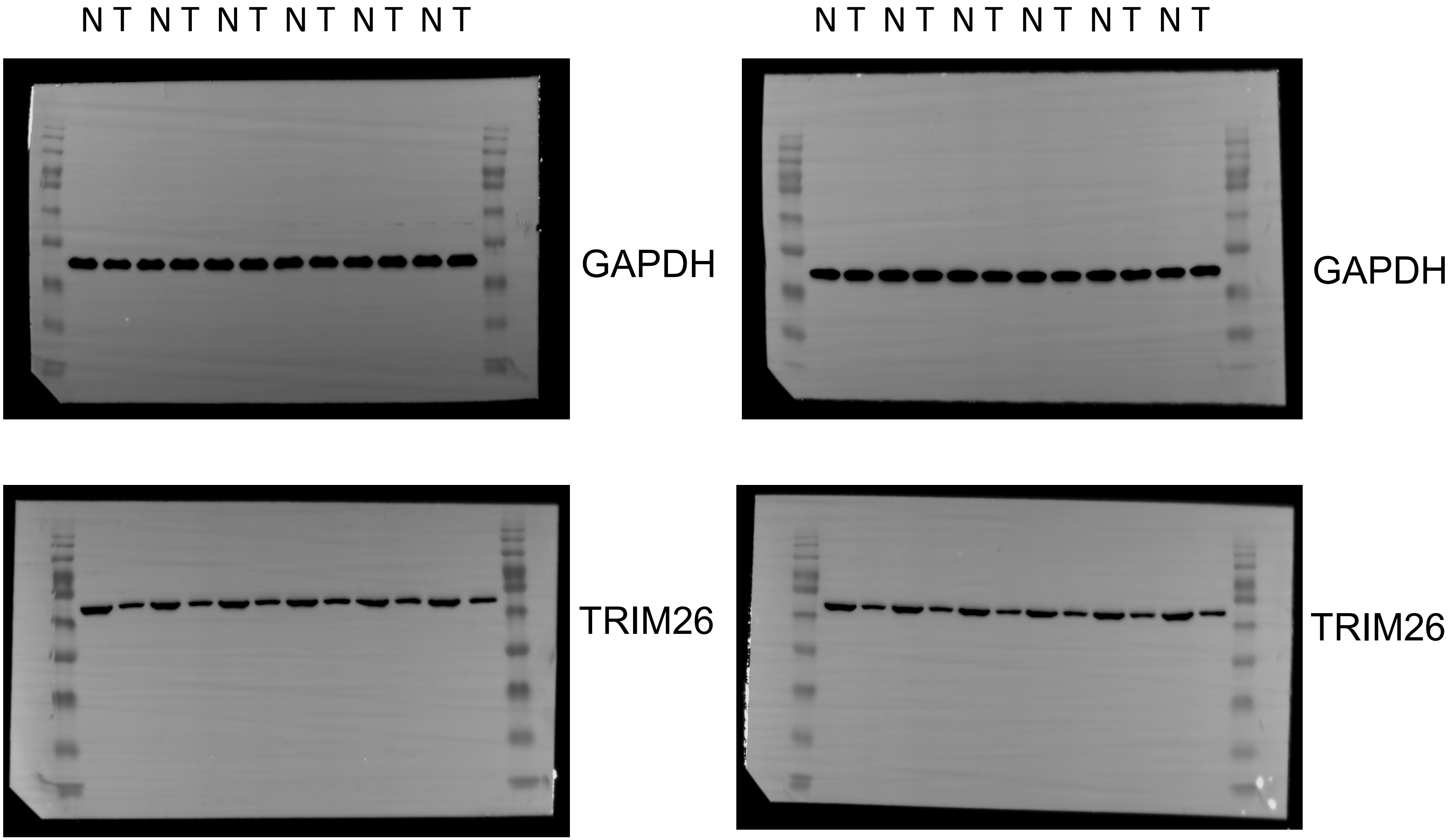

Fig. 2B

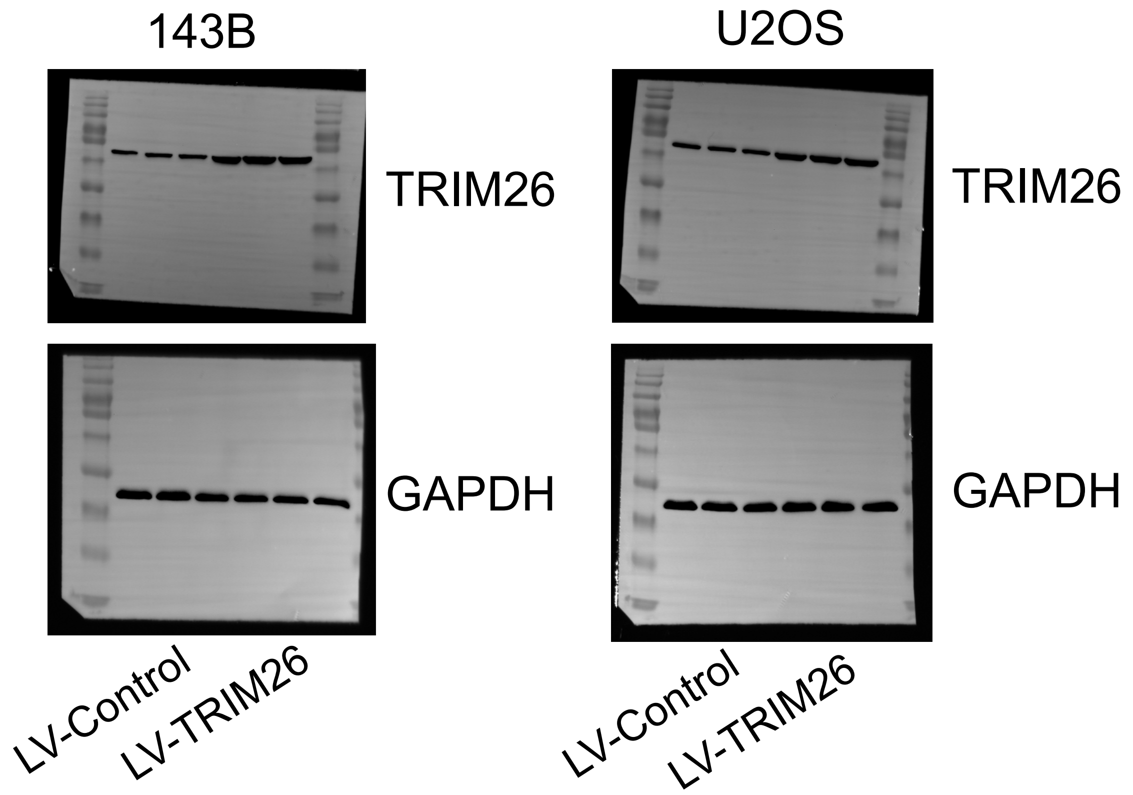
143B
U2OS
TRIM26
TRIM26
GAPDH
GAPDH
LV-Control
LV-TRIM26
LV-Control
LV-TRIM26

Fig. 2J

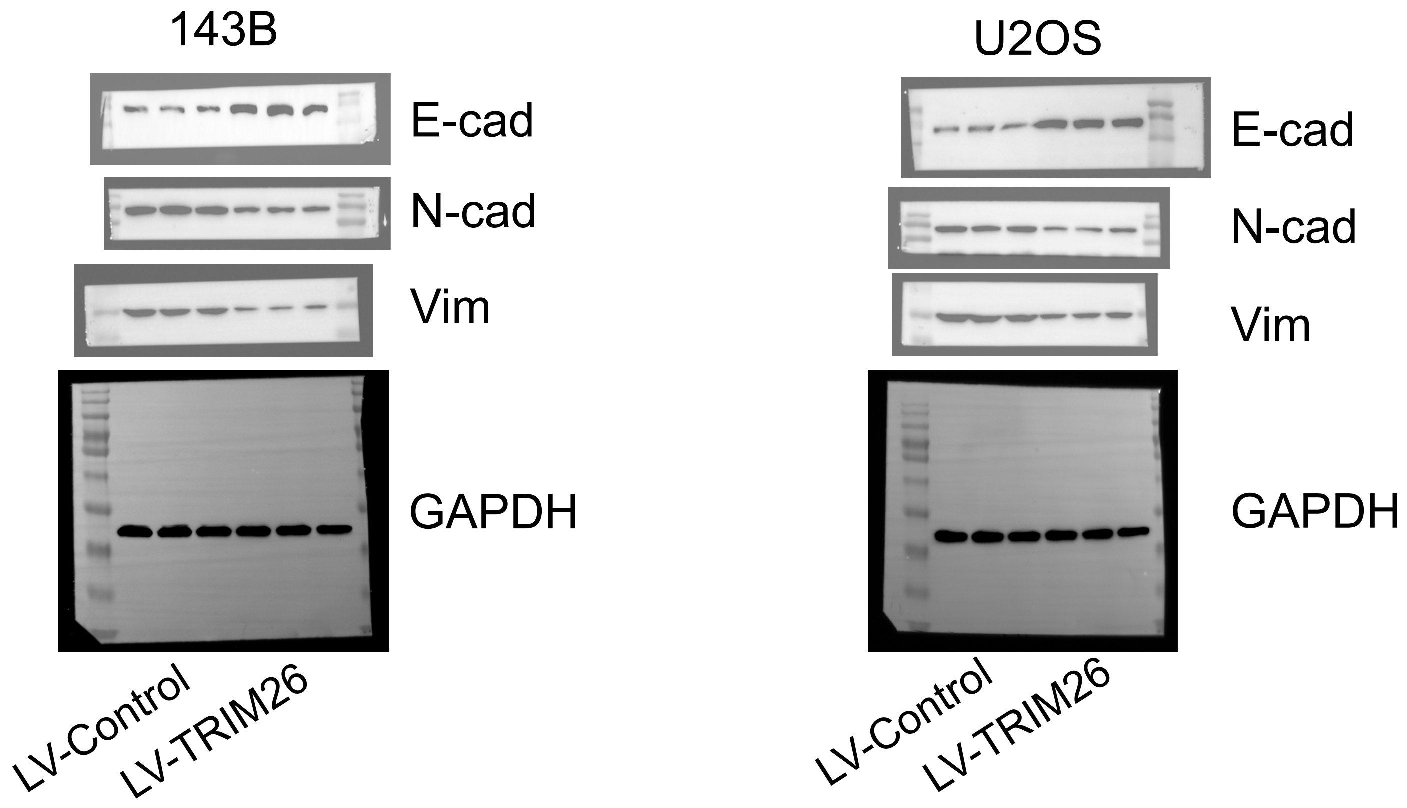
143B
U2OS
E-cad
N-cad
Vim
GAPDH
E-cad
N-cad
Vim
GAPDH
LV-Control
LV-TRIM26
LV-Control
LV-TRIM26

Fig. 3B

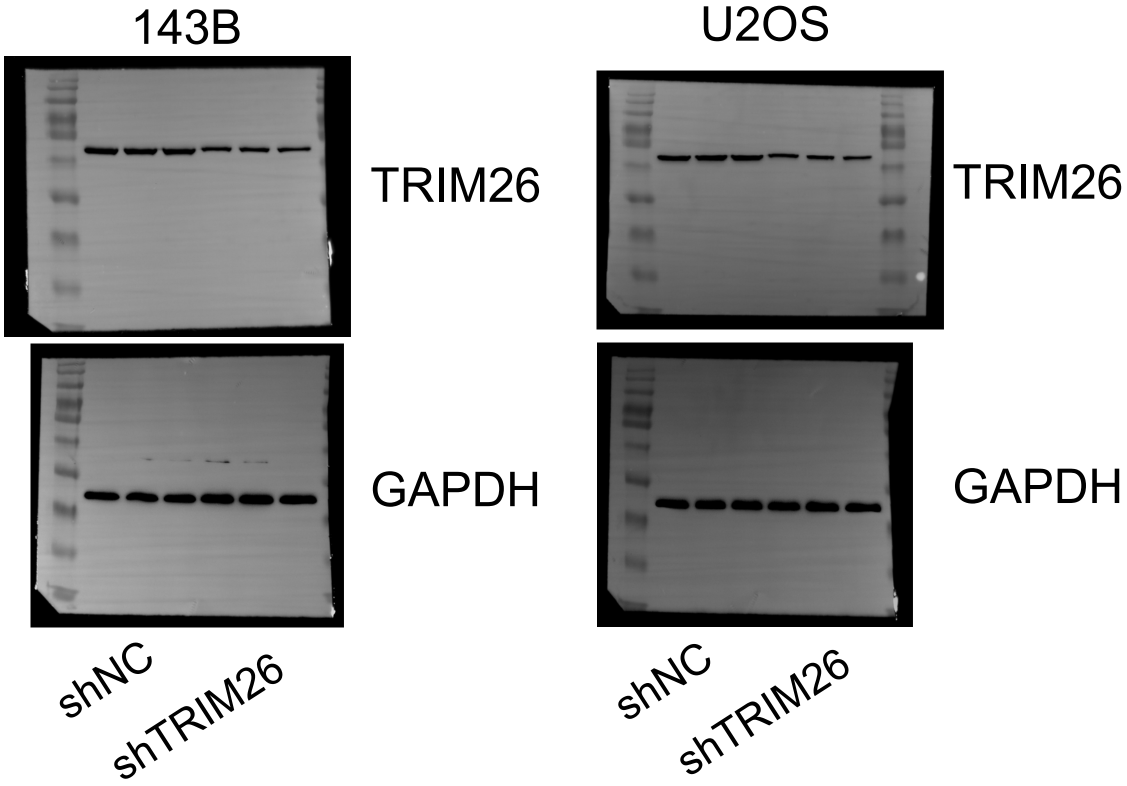
143B
U2OS
TRIM26
TRIM26
GAPDH
GAPDH
shNC
shTRIM26
shNC
shTRIM26

Fig. 3J

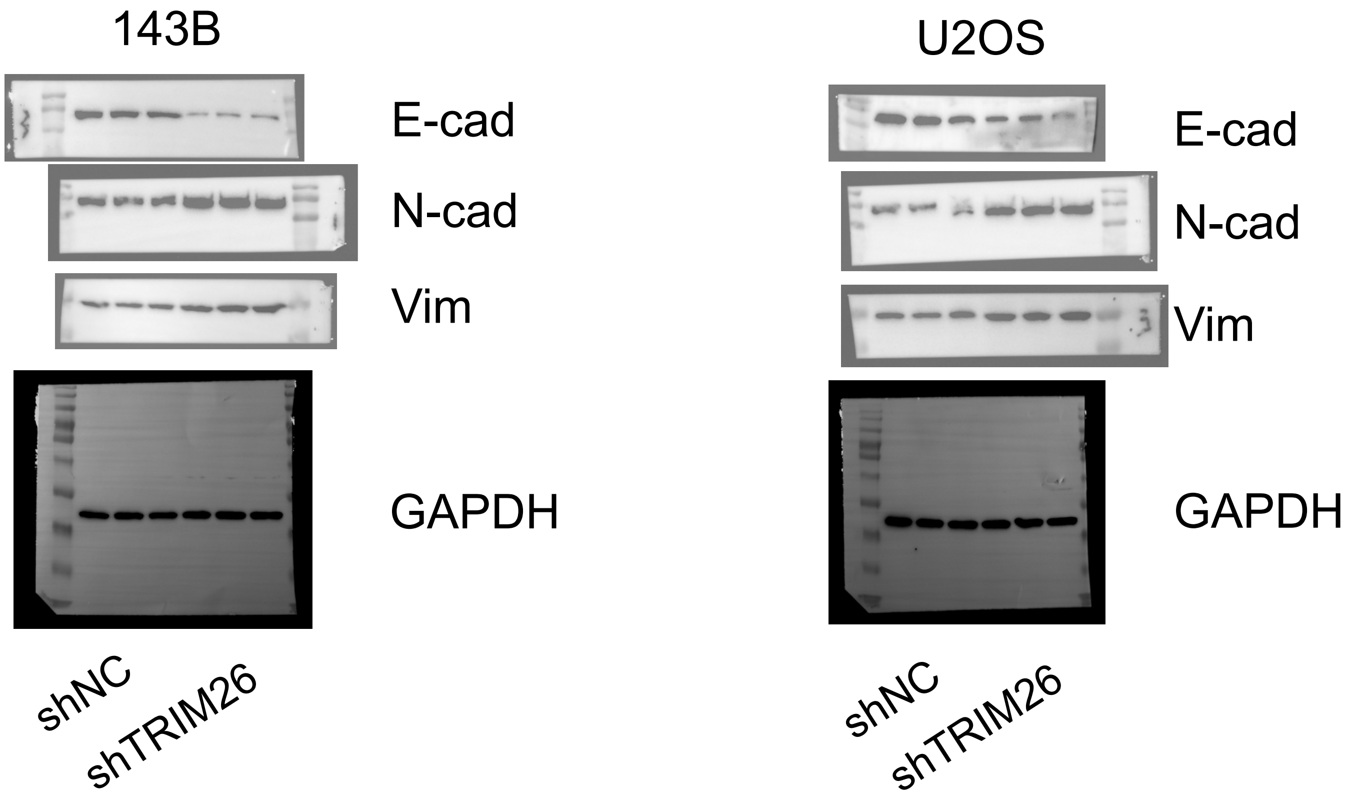
143B
E-cad
N-cad
Vim
GAPDH
shNC
shTRIM26
U2OS
E-cad
N-cad
Vim
GAPDH
shNC
shTRIM26

Fig. 4D

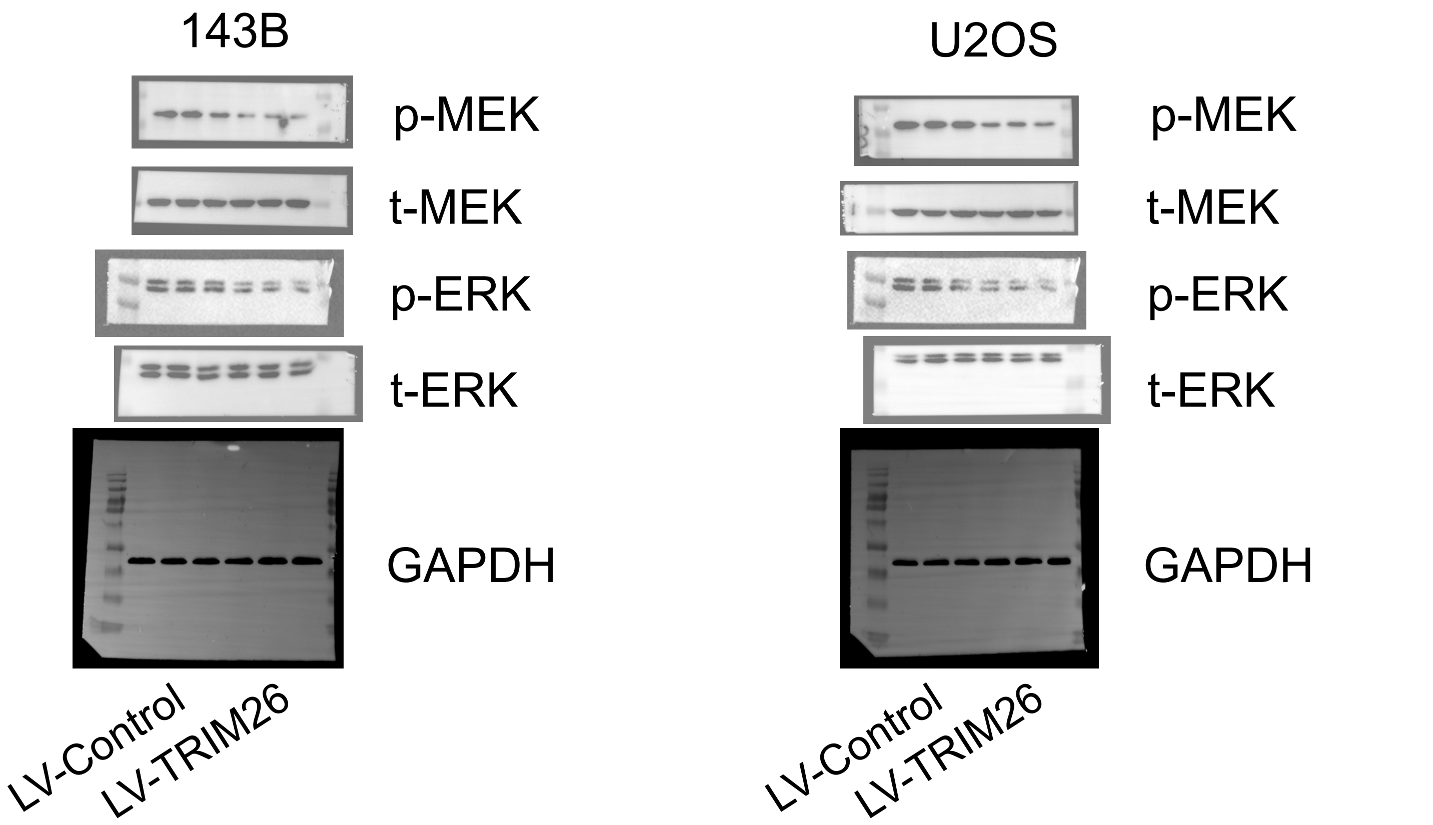

Fig. 4G

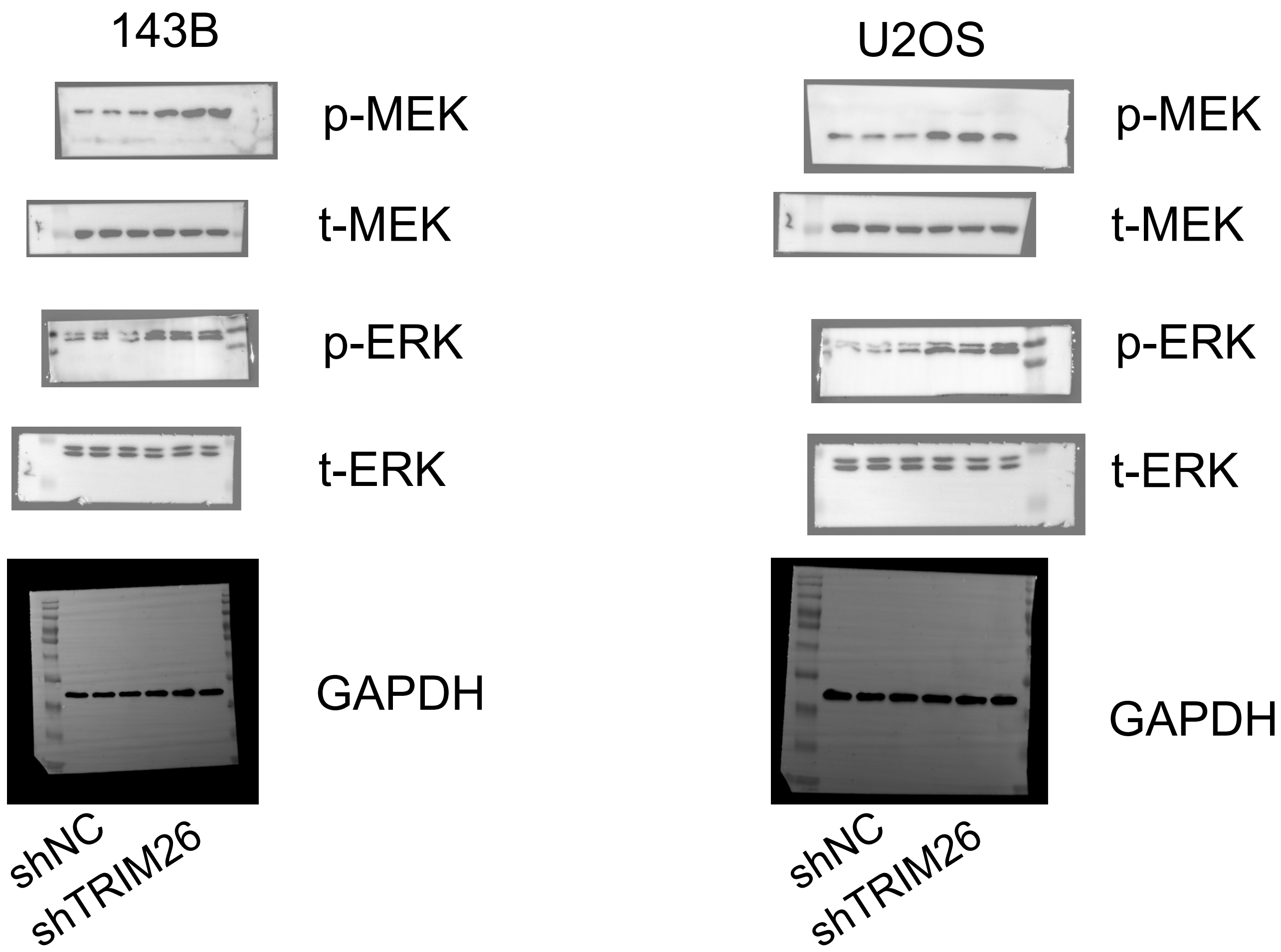
143B
U2OS
p-MEK
t-MEK
p-ERK
t-ERK
GAPDH
p-MEK
t-MEK
p-ERK
t-ERK
GAPDH
shNC
shTRIM26
shNC
shTRIM26

Fig. 5A

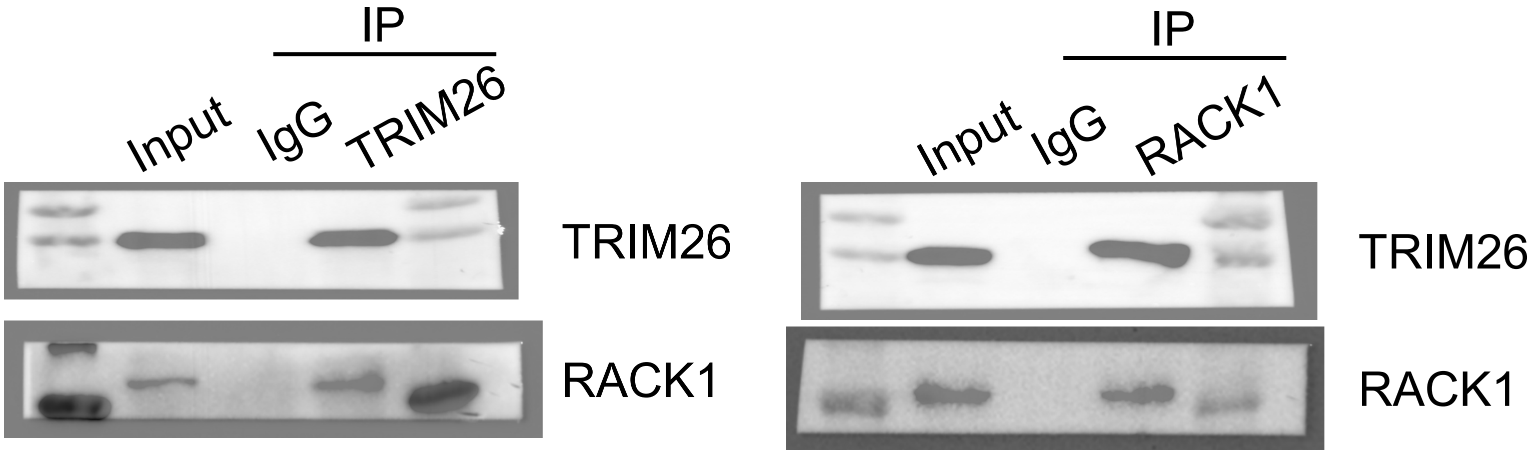

IP
Input
IgG
TRIM26
TRIM26
RACK1
IP
Input
IgG
RACK1
TRIM26
RACK1

Fig. 5B

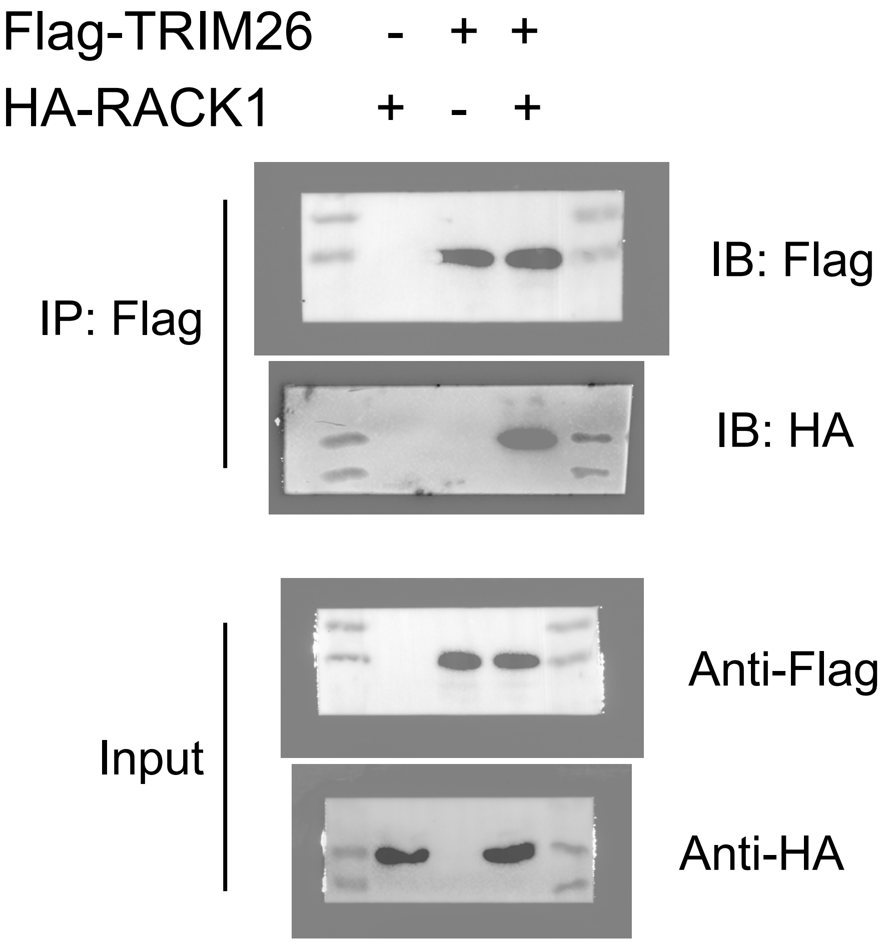

Flag-TRIM26 - + +
HA-RACK1 + - +
IP: Flag
IB: Flag
IB: HA
Input
Anti-Flag
Anti-HA

Fig. 5E

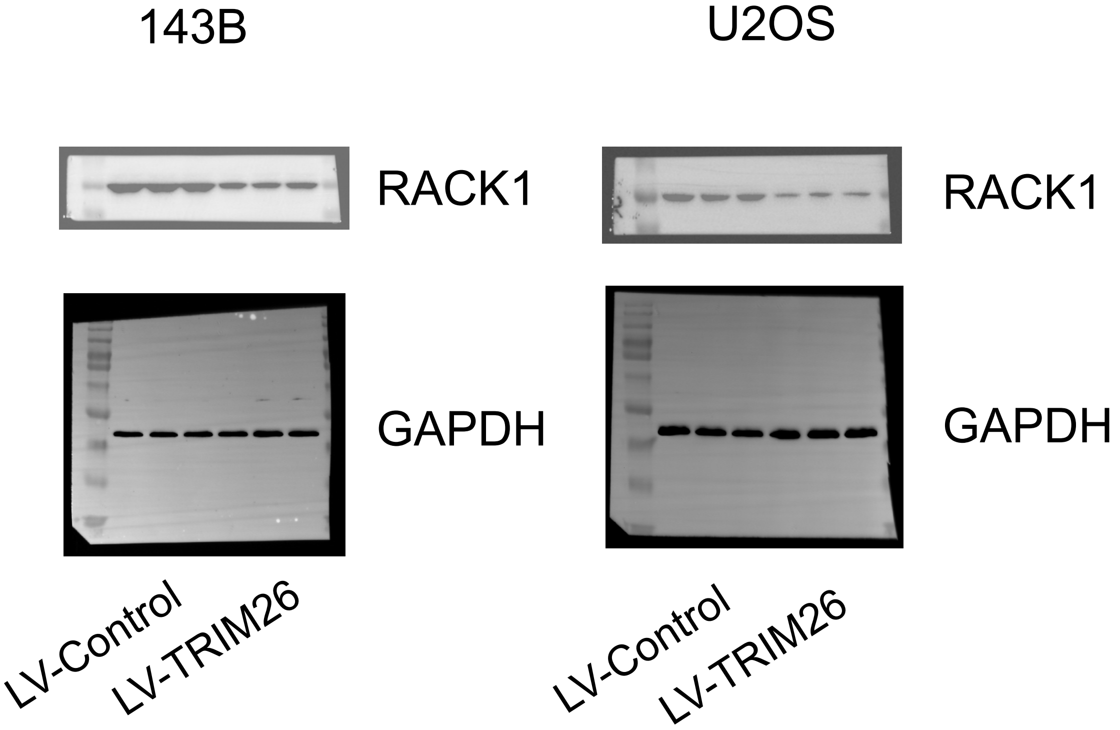
143B
U2OS
RACK1
RACK1
GAPDH
GAPDH
LV-Control
LV-TRIM26
LV-Control
LV-TRIM26

Fig. 5G

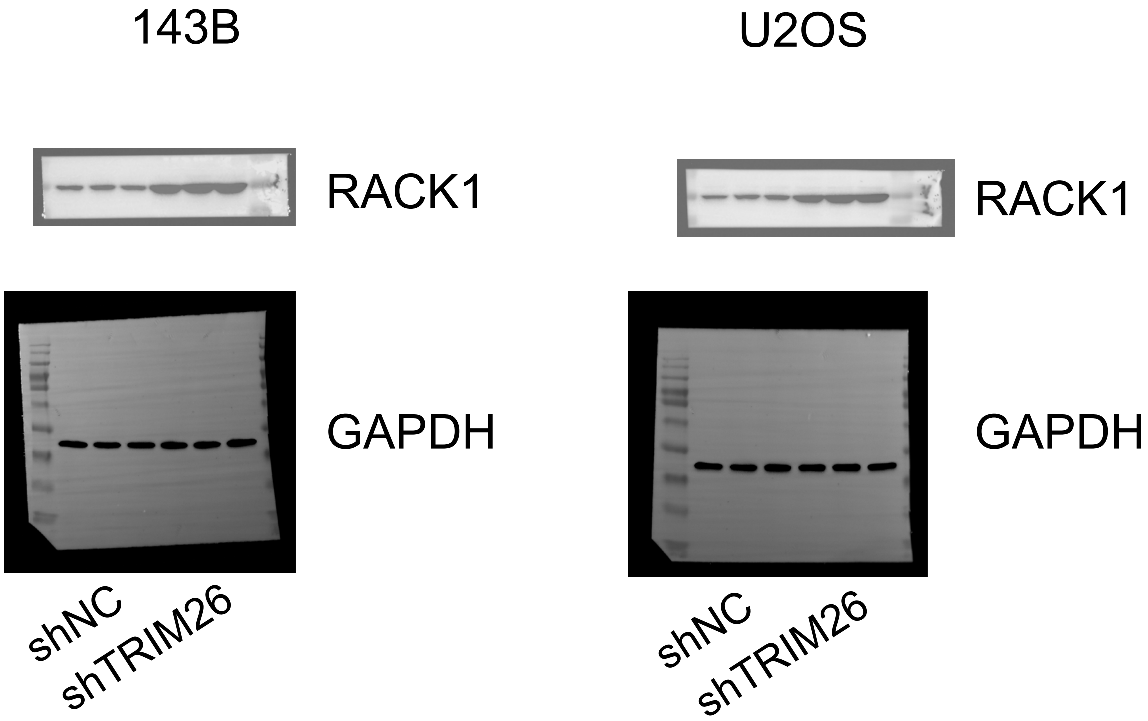
143B
U2OS
RACK1
RACK1
GAPDH
GAPDH
shNC
shTRIM26
shNC
shTRIM26

Fig. 5I

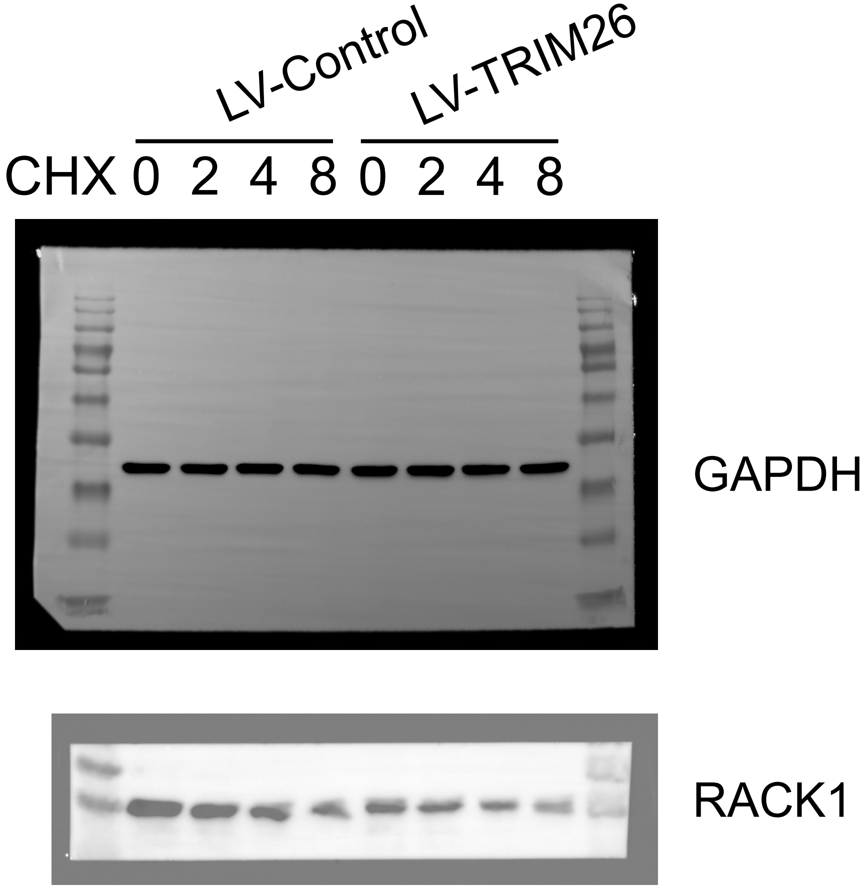

Fig. 5K

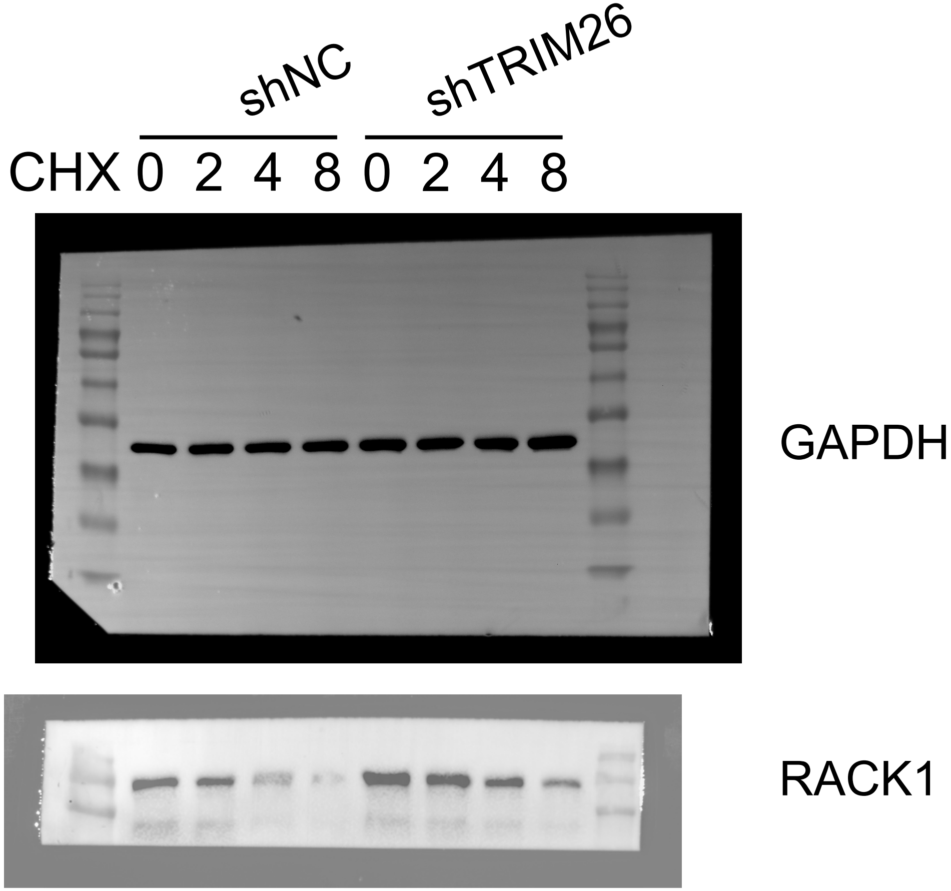

Fig. 5M-N

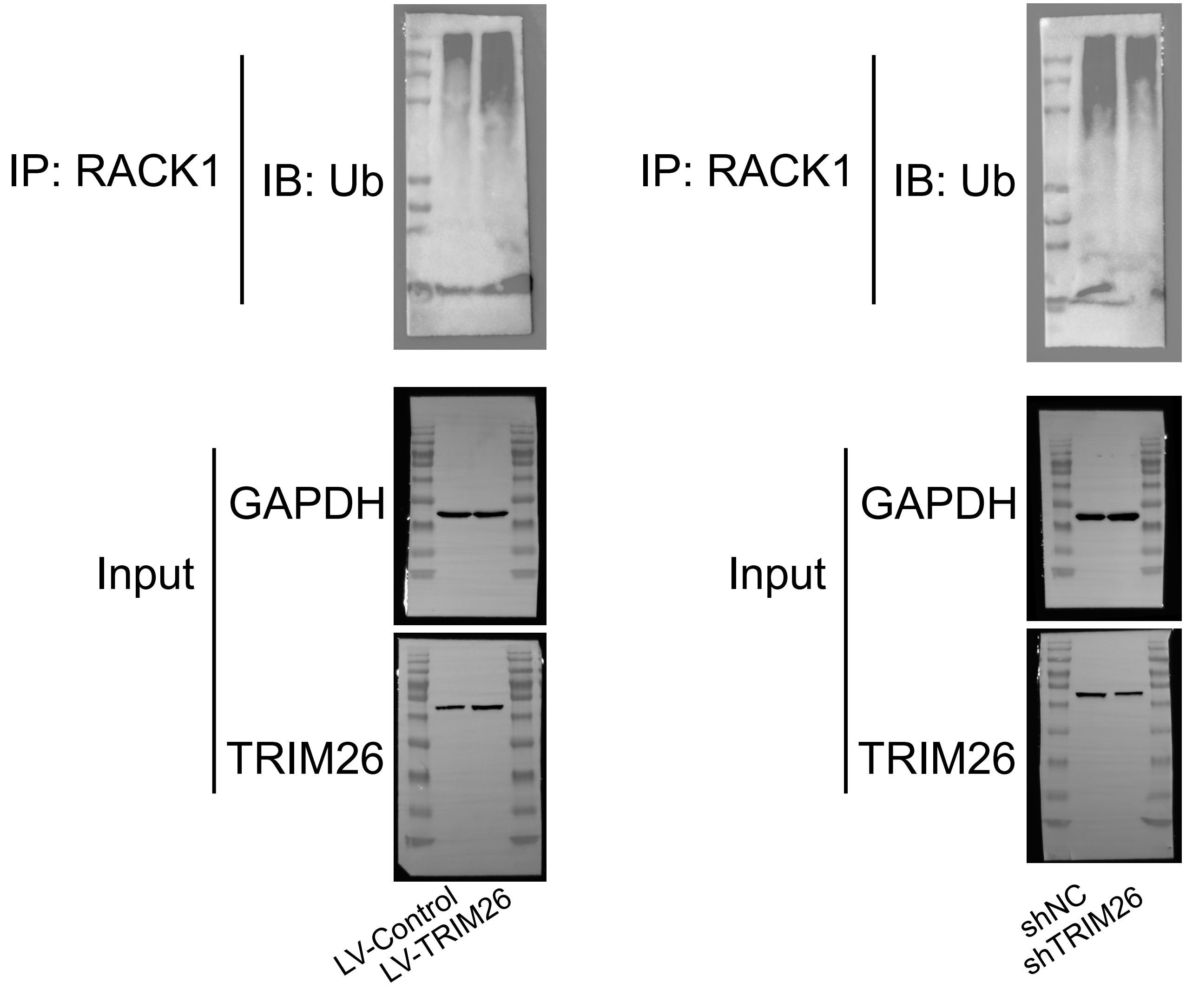
IP: RACK1
IB: Ub
Input
GAPDH
TRIM26
LV-Control
LV-TRIM26
IP: RACK1
IB: Ub
Input
GAPDH
TRIM26
shNC
shTRIM26

Fig. 6A

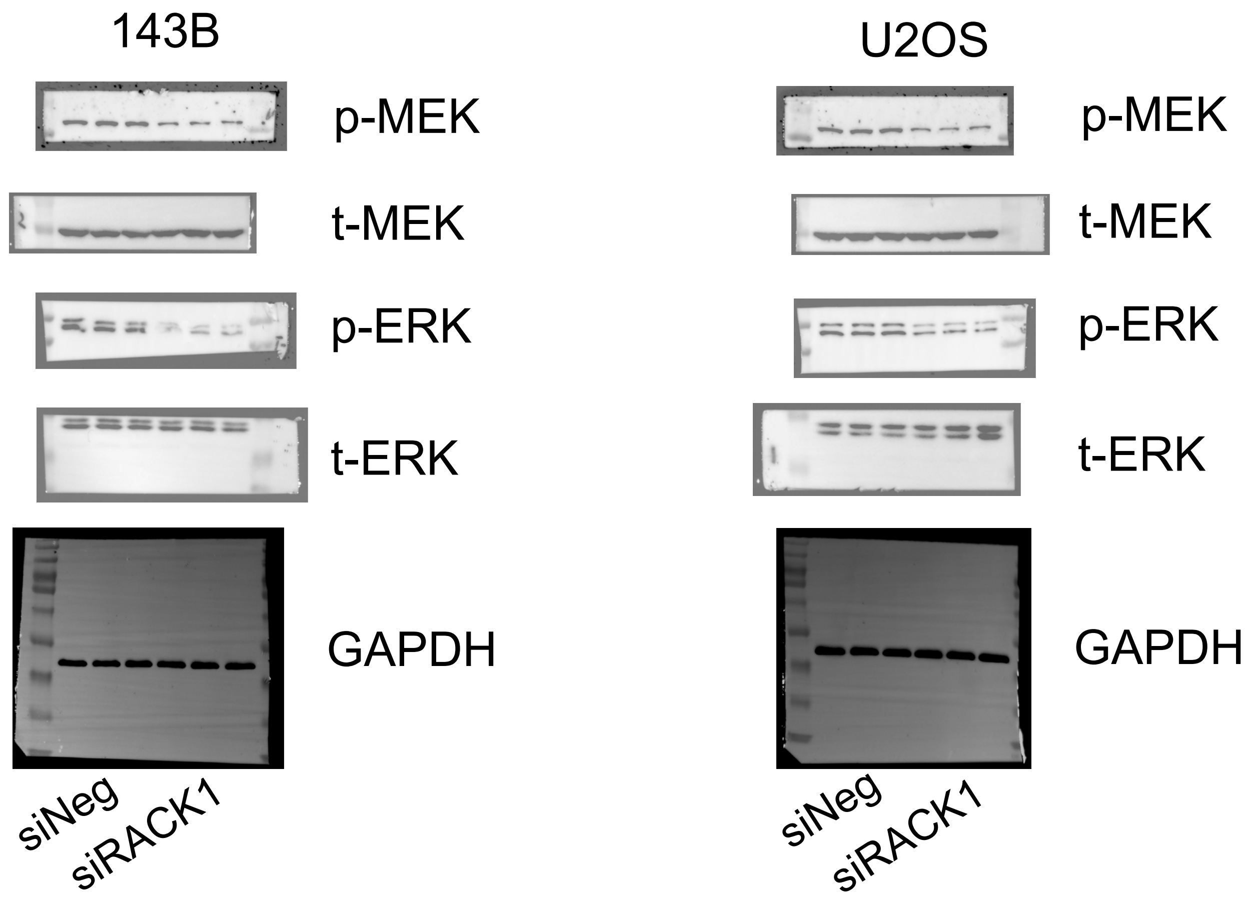
143B
U2OS
p-MEK
t-MEK
p-ERK
t-ERK
GAPDH
p-MEK
t-MEK
p-ERK
t-ERK
GAPDH
siNeg
siRACK1
siNeg
siRACK1

Fig. 6D

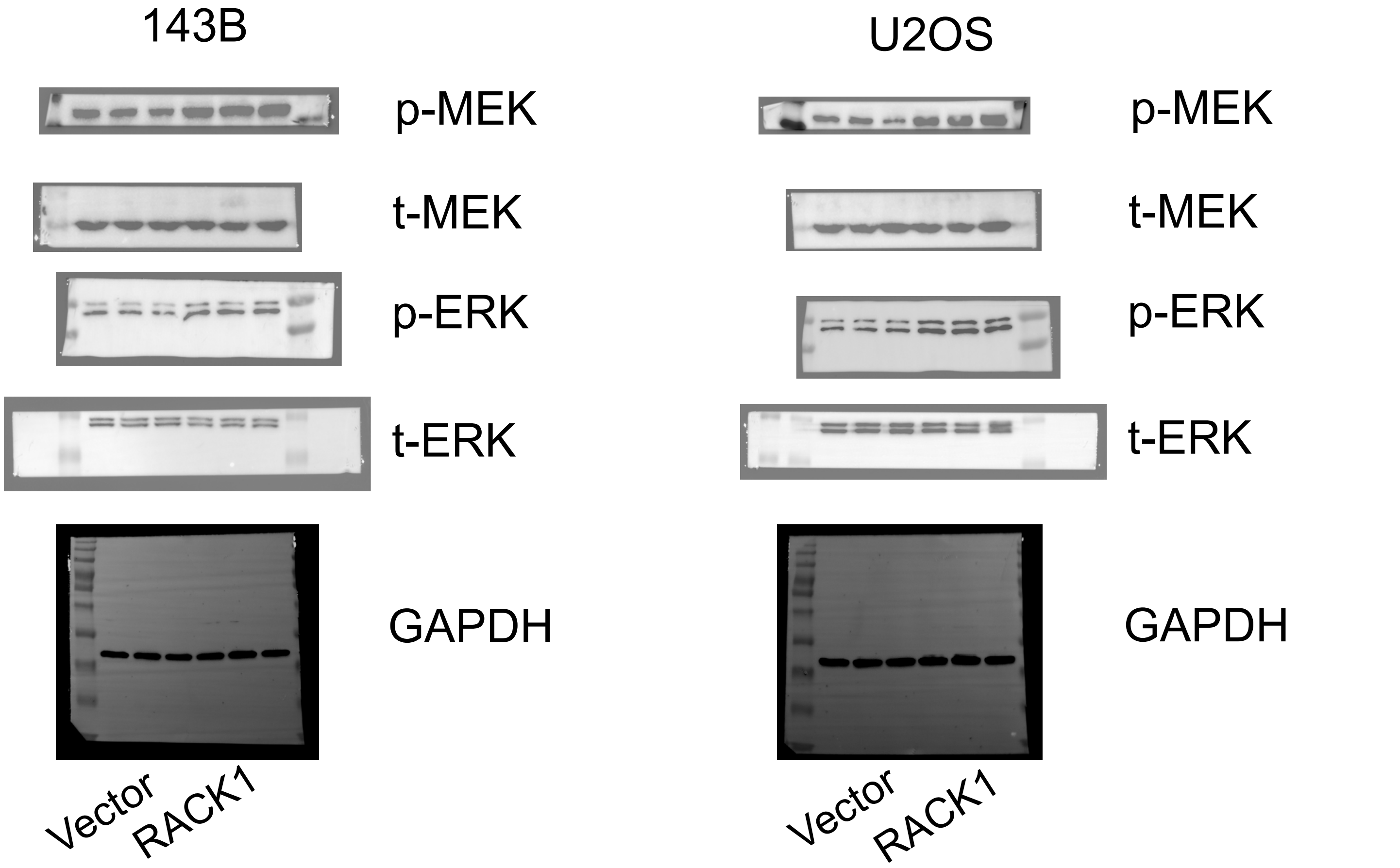
143B
U2OS
p-MEK
t-MEK
p-ERK
t-ERK
GAPDH
p-MEK
t-MEK
p-ERK
t-ERK
GAPDH
Vector
RACK1
Vector
RACK1

Fig. 6G

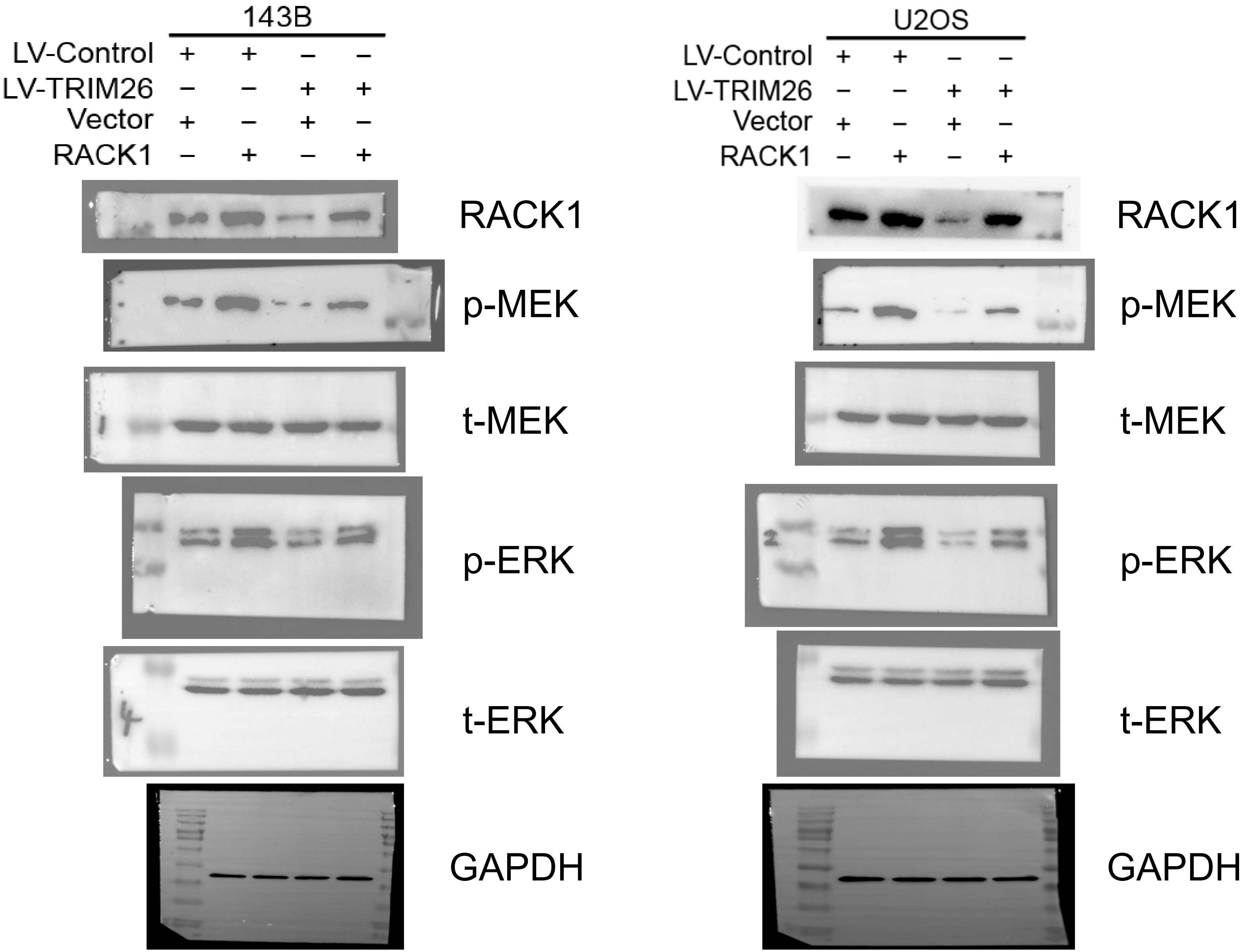
143B
LV-Control + + − −
LV-TRIM26 − − + +
Vector + − + −
RACK1 − + − +
RACK1
p-MEK
t-MEK
p-ERK
t-ERK
GAPDH
U2OS
LV-Control + + − −
LV-TRIM26 − − + +
Vector + − + −
RACK1 − + − +
RACK1
p-MEK
t-MEK
p-ERK
t-ERK
GAPDH

Fig. 6J

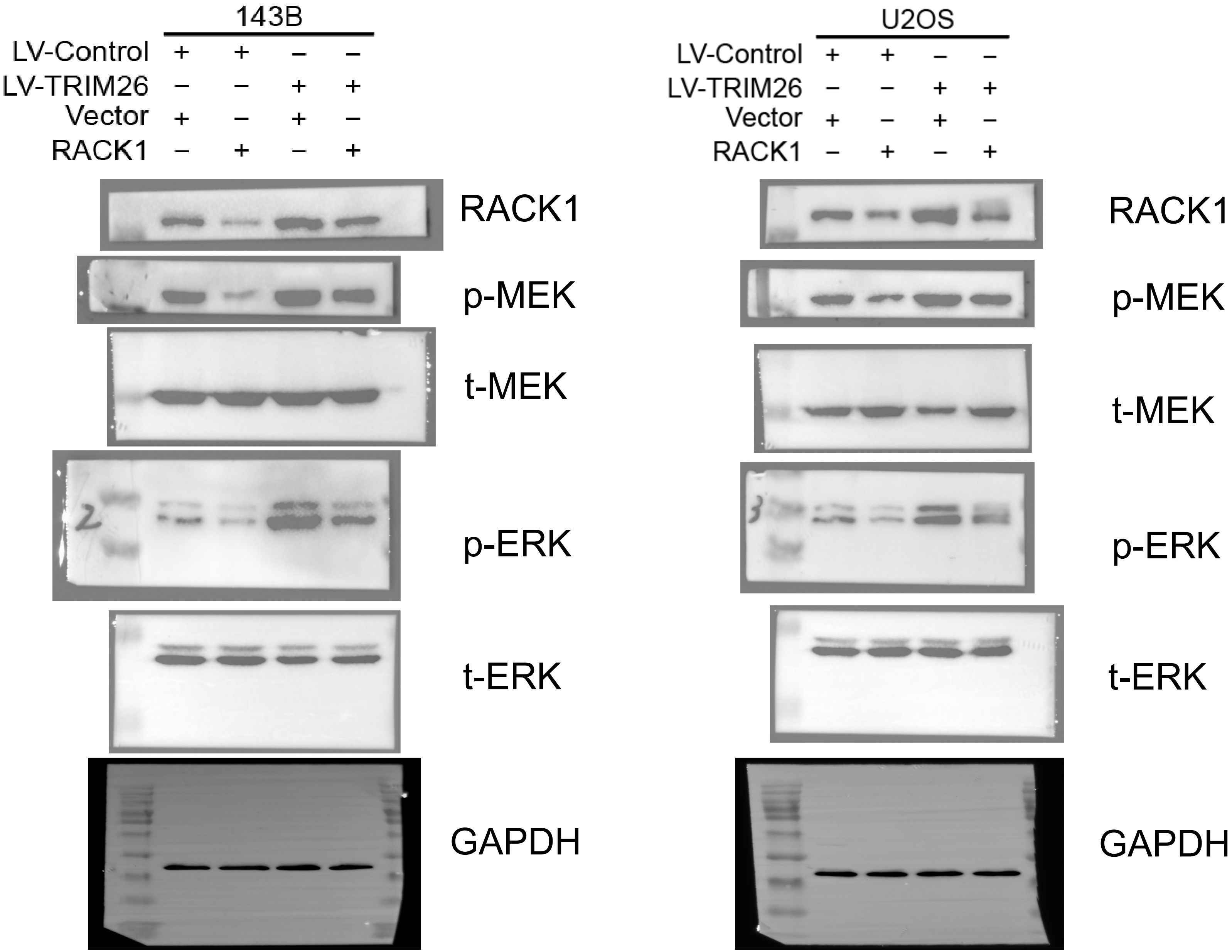
143B
LV-Control + + − −
LV-TRIM26 − − + +
Vector + − + −
RACK1 − + − +
RACK1
p-MEK
t-MEK
p-ERK
t-ERK
GAPDH
U2OS
LV-Control + + − −
LV-TRIM26 − − + +
Vector + − + −
RACK1 − + − +
RACK1
p-MEK
t-MEK
p-ERK
t-ERK
GAPDH

Fig. 7D

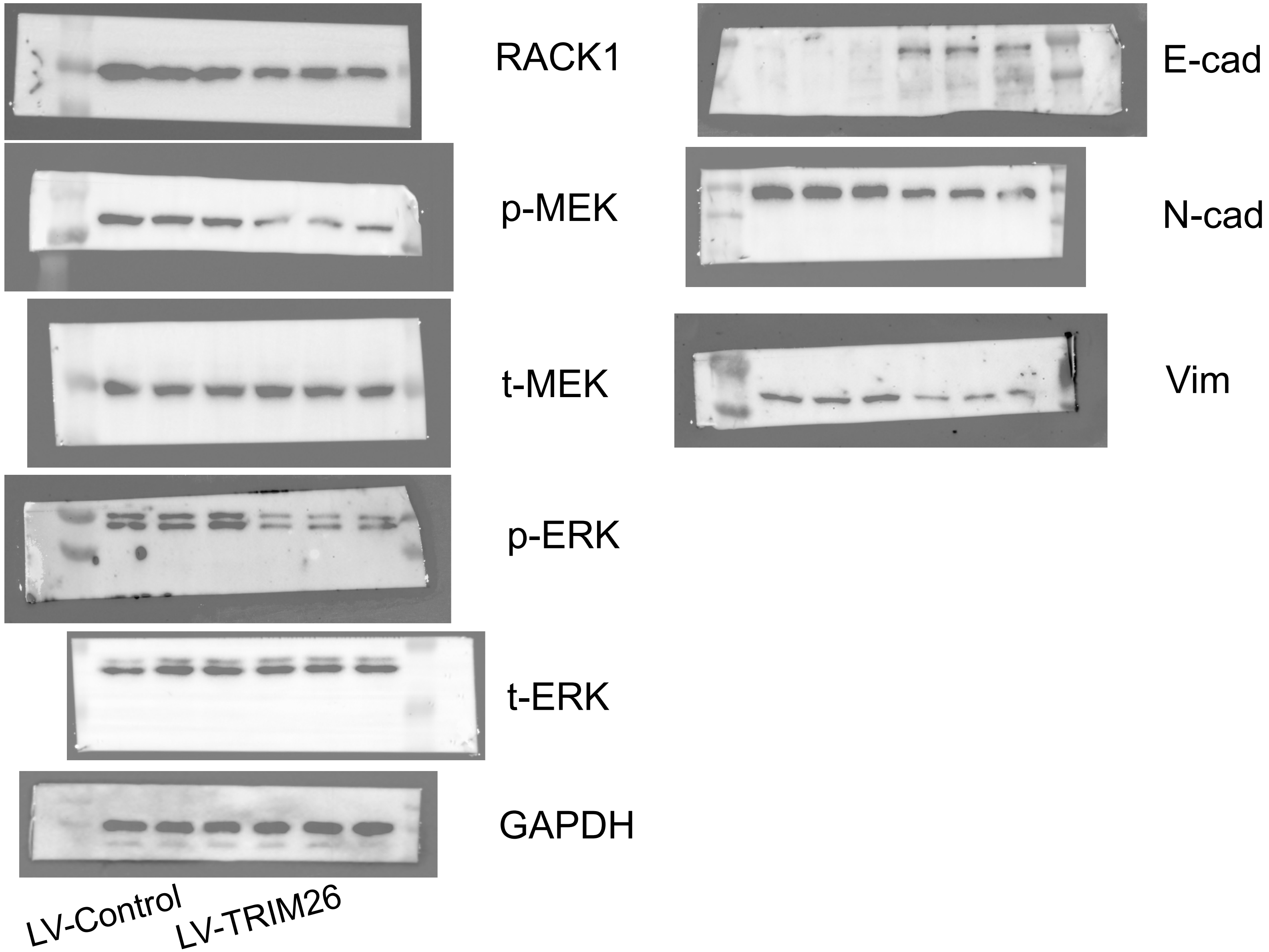
RACK1
p-MEK
t-MEK
p-ERK
t-ERK
GAPDH
LV-Control
LV-TRIM26
E-cad
N-cad
Vim
